# Functional genomics study of acute heat stress response in the small yellow follicles of layer-type chickens

**DOI:** 10.1038/s41598-017-18335-5

**Published:** 2018-01-22

**Authors:** Chuen-Yu Cheng, Wei-Lin Tu, Chao-Jung Chen, Hong-Lin Chan, Chih-Feng Chen, Hsin-Hsin Chen, Pin-Chi Tang, Yen-Pai Lee, Shuen-Ei Chen, San-Yuan Huang

**Affiliations:** 1 Department of Animal Science, National Chung Hsing University, Taichung, Taiwan; 2 Department of Medical Research, Proteomic Core Laboratory, China Medical University Hospital, Taichung, Taiwan; 3 Graduate Institute of Integrated Medicine, China Medical University, Taichung, Taiwan; 4 Institute of Bioinformatics and Structural Biology, National Tsing Hua University, Hsinchu, Taiwan; 5 Department of Medical Sciences, National Tsing Hua University, Hsinchu, Taiwan; 6 Agricultural Biotechnology Center, National Chung Hsing University, Taichung, Taiwan; 7 Center for the Integrative and Evolutionary Galliformes Genomics, iEGG Center, National Chung Hsing University, Taichung, Taiwan; 8 Research Center for Sustainable Energy and Nanotechnology, National Chung Hsing University, Taichung, Taiwan

## Abstract

This study investigated global gene and protein expression in the small yellow follicle (SYF; 6–8 mm in diameter) tissues of chickens in response to acute heat stress. Twelve 30-week-old layer-type hens were divided into four groups: control hens were maintained at 25 °C while treatment hens were subjected to acute heat stress at 36 °C for 4 h without recovery, with 2-h recovery, and with 6-h recovery. SYFs were collected at each time point for mRNA and protein analyses. A total of 176 genes and 93 distinct proteins with differential expressions were identified, mainly associated with the molecular functions of catalytic activity and binding. The upregulated expression of heat shock proteins and peroxiredoxin family after acute heat stress is suggestive of responsive machineries to protect cells from apoptosis and oxidative insults. In conclusion, both the transcripts and proteins associated with apoptosis, stress response, and antioxidative defense were upregulated in the SYFs of layer-type hens to alleviate the detrimental effects by acute heat stress. However, the genomic regulations of specific cell type in response to acute heat stress of SYFs require further investigation.

## Introduction

Heat stress affects the secretion of sexual hormones, such as prolactin, luteinizing hormone, and follicle-stimulating hormone, which might reduce the fertility of female domestic animals^1–5^. In addition, heat stress hampers the development of ovarian follicles and tends to cause ovarian regression^6–10^. The production characteristics of layer-type hens, including the body weight, egg-laying rate, egg weight, egg shell thickness, and feed efficiency, are decreased under heat stress conditions^11–15^. Small yellow follicles (SYFs) reside in a crucial prehierarchical stage and form a pool, from which a single follicle is selected into the hierarchy every day and destined for ovulation^16–18^.

Heat stress in livestock is a prevalent challenge in most subtropical and tropical areas, particularly for the popular commercial strains of domestic animals bred in temperate countries. Tropical country chickens are native, slow-growing breeds which are adapted to the local climates and thus tend to exhibit better thermotolerance than do exotic commercial breeds^19^. A global functional genomic study to profile gene and protein expressions of follicles in response to acute heat stress in layer-type tropical native chickens has never been reported. A layer-type strain of Taiwan country chickens (TCC, L2 strain) has been bred for high egg production for more than 30 generations^20^ and thus serves as a good model to study the thermal response of reproductive function in tropical fowls. This study investigated the functional genomic changes of SYFs in response to acute heat stress in layer-type tropical chickens.

## Results

### Effect of acute heat stress on physiological parameters

**Figure 1:**
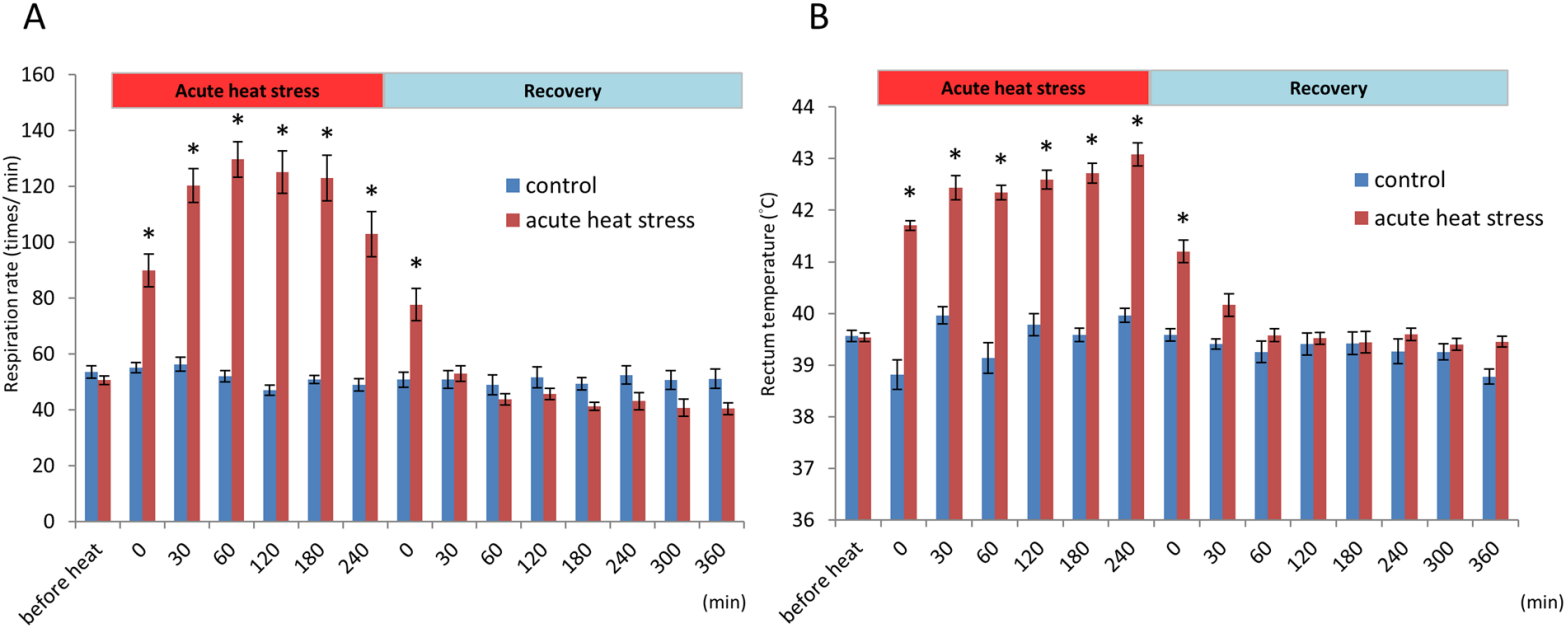
Respiratory rate (**A**) and body temperature (**B**) of acute heat-stressed and control hens (layer-type L2 strain Taiwan country chickens) during the heat stress and recovery periods. The asterisk indicate the values differ significantly between the heat-stressed and control groups (P < 0.05).

Acute heat stress at 36 °C significantly increased the respiratory rate and body temperature immediately after treatment initiation (P < 0.05; Fig. 1). The hens began panting 30 min after heat stress, and the body temperatures significantly increased in the heat-stressed groups during the heat stress period (P < 0.05).

### Gene expression and annotation of differentially expressed genes in the SYFs after acute heat stress

**Figure 2:**
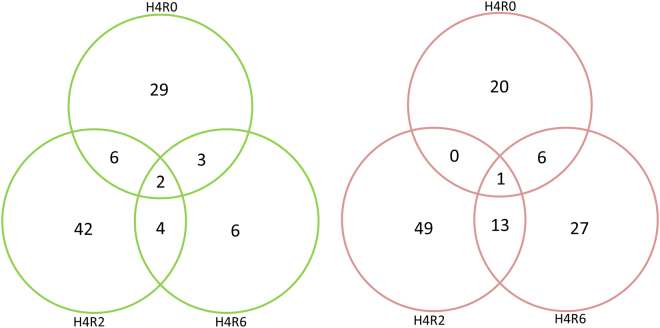
Venn diagram analysis of 92 upregulated (**A**) and 116 downregulated (**B**) probes in the SYFs of layer-type L2 strain Taiwan country chickens which underwent 36 °C acute heat stress for 4 h and recovery for 0, 2, and 6 h. There were 202 probes significantly differential expressed (over 2 fold change compared with control group) in microarray analysis in SYFs after acute heat stress. H4R0, without recovery after heat stress; H4R2, recovery for 2 h after heat stress; H4R6, recovery for 6 h after heat stress.

**Figure 3:**
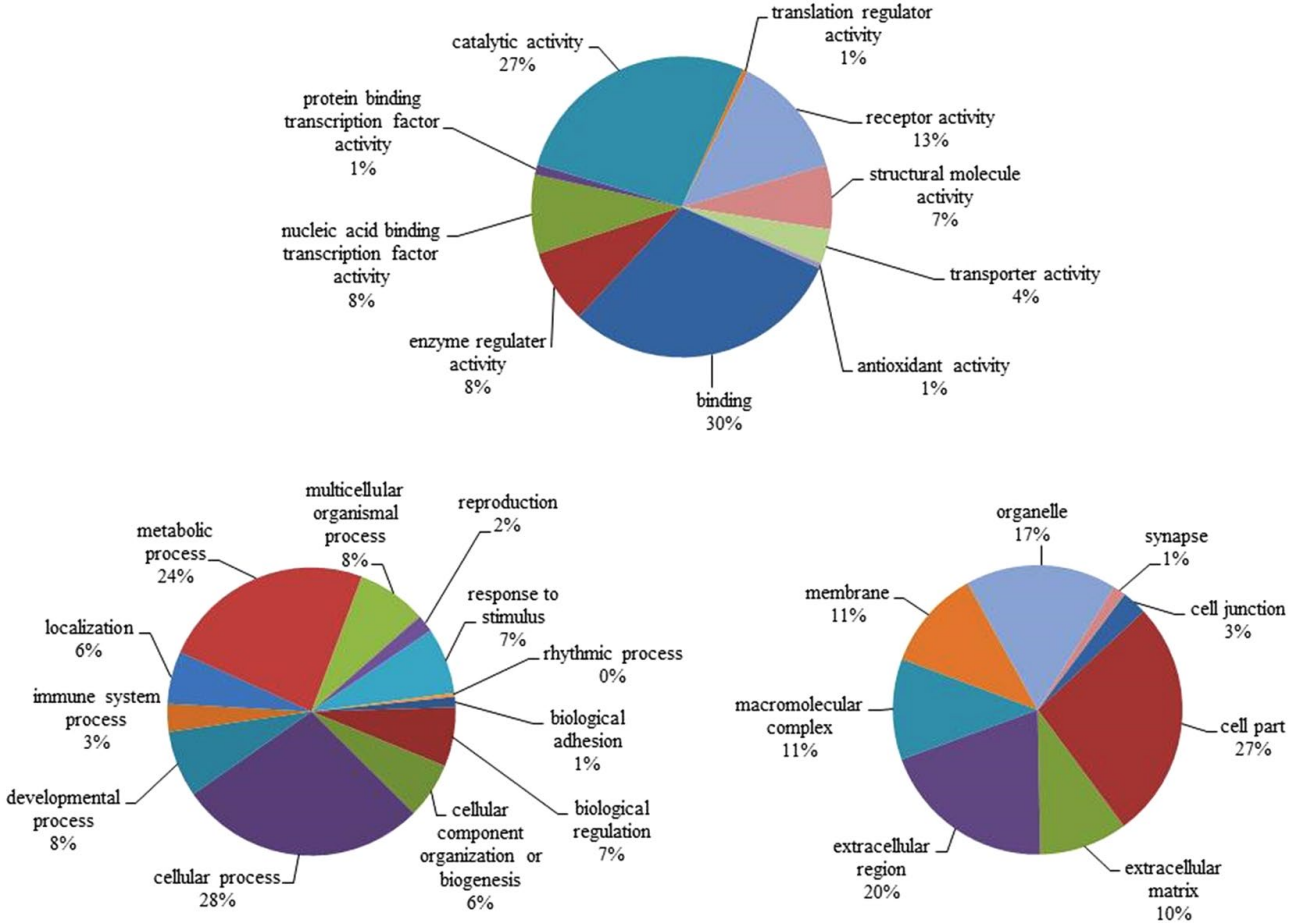
Pie charts showing the classification of differentially expressed genes in the SYFs of layer-type L2 strain Taiwan country chickens who underwent 36 °C acute heat stress for 4 h and recovery for 0, 2, and 6 h, stratified by molecular functions (**A**), biological processes (**B**), and cellular components (**C**).

The transcription profiles of the SYFs of CTL and heat-stressed hens allowed 0, 2, and 6 h recovery were analyzed using the chicken 44 K oligo microarray. A total of 176 genes in the SYFs showed differential expressions after heat stress with the abundance cut off at two-fold changes (P < 0.05; Supplementary Table S1). In the H4R0, H4R2, and H4R6 groups, 56, 103, and 56 gene expressions differed from CTL hens, respectively (Fig. 2; Supplementary Table S1), with 32, 50, and 14 gene transcripts upregulated but 24, 53, and 42 genes downregulated. Ache and Ca5b showed consistently higher expressions but LOC100859660 was downregulated at all recovery times after the heat exposure. Gene ontology annotation revealed that most of the differentially expressed genes participated in cellular processes (28%), metabolic processes (24%), developmental processes (8%), and multicellular organismal processes (8%; Fig. 3). In molecular function, the genes were mainly categorized by binding (30%), catalytic activity (27%), receptor activity (13%), and nucleic acid binding transcription factor activity (8%). In terms of cellular components, these genes were primarily located in the cell (27%), extracellular region (20%), organelles (17%), and membranes (11%).

### Validation of representative differential gene expressions in the SYFs after acute heat stress

**Figure 4:**
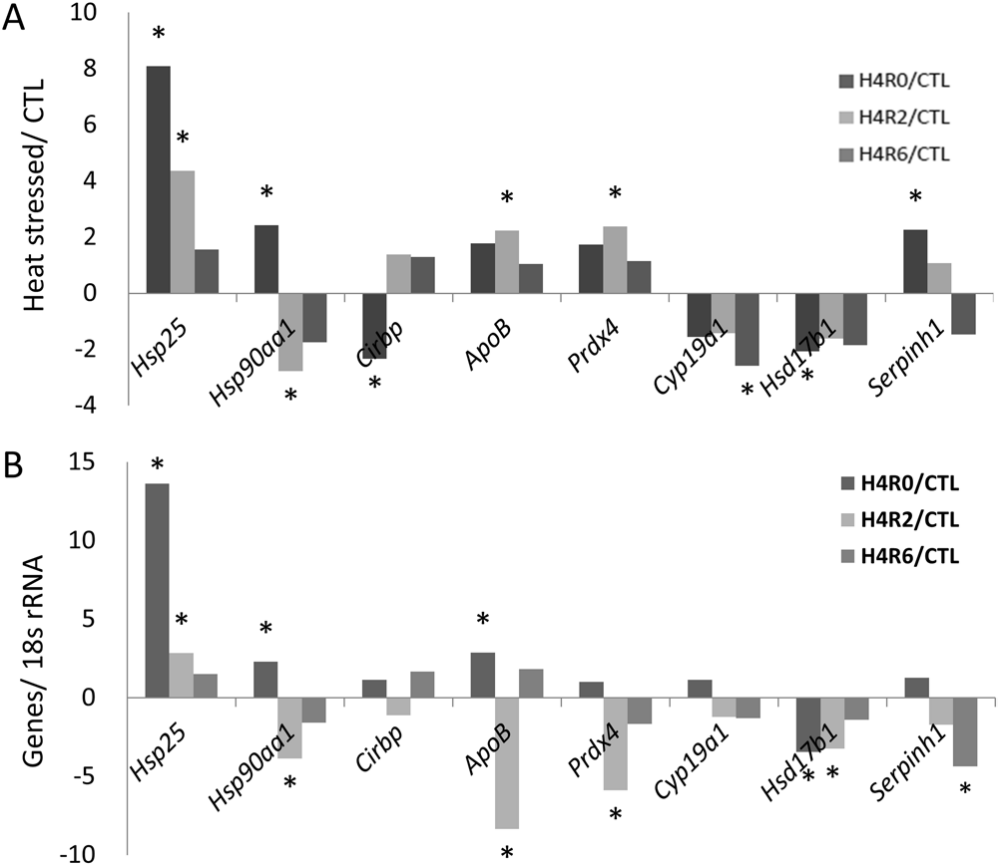
Multiples of changes of significantly differentially expressed genes in the SYFs of layer-type L2 strain Taiwan country chickens after acute heat stress were determined through microarray (**A**) and quantitative reverse transcription polymerase chain reaction analysis (**B**). The cutoff value for the differentially expressed genes was set to a two-fold or higher change.

Eight differentially expressed genes observed in the microarray analysis were validated through qRT-PCR (Fig. 4). The expression patterns of Hsp25, Hsp90aa1, and Hsd17b1 coincided with the results of microarray analysis, whereas ApoB, Prdx4, and Serpinh1 expressions behaved controversially, in particular for the H4R2 group in which they all seemed to be downregulated. Cirbp and Cyp19a1 did not show significant difference after acute heat treatment in qRT-PCR analysis. However, Cirbp and Cyp19a1 were downregulated in the H4R0 and H4R6 groups, respectively, in microarray analysis.

### Comparison of protein profiles and identification of differentially expressed proteins in SYFs after acute heat stress

**Figure 5:**
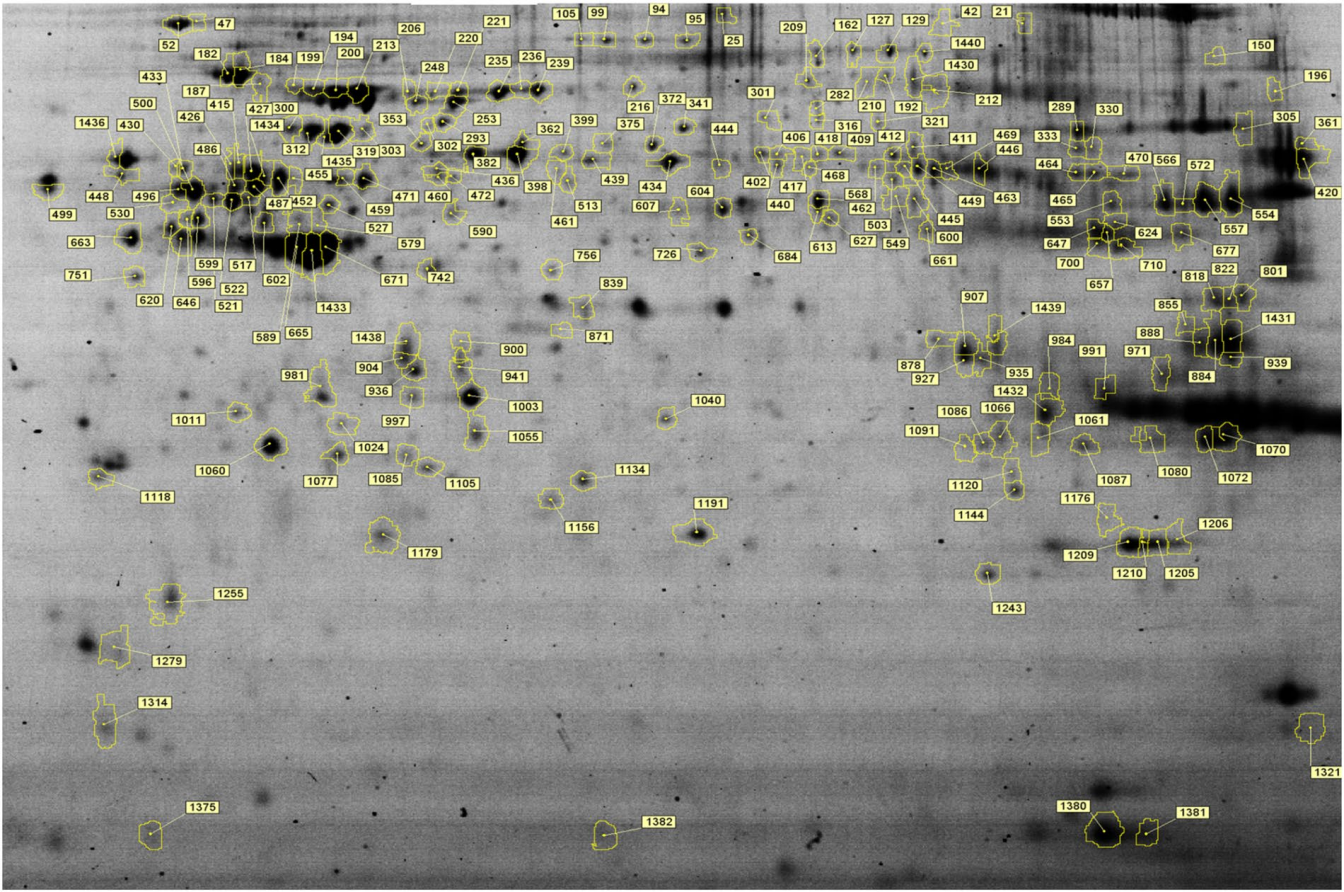
2D-DIGE protein profiles of the SYFs of layer-type L2 strain Taiwan country chickens. The numbers show the protein spots with significantly higher expressions (≥1.3, P < 0.05) in the control group or heat-stressed groups. The raw profiles are showed in supplementary Fig. S1.

**Figure 6:**
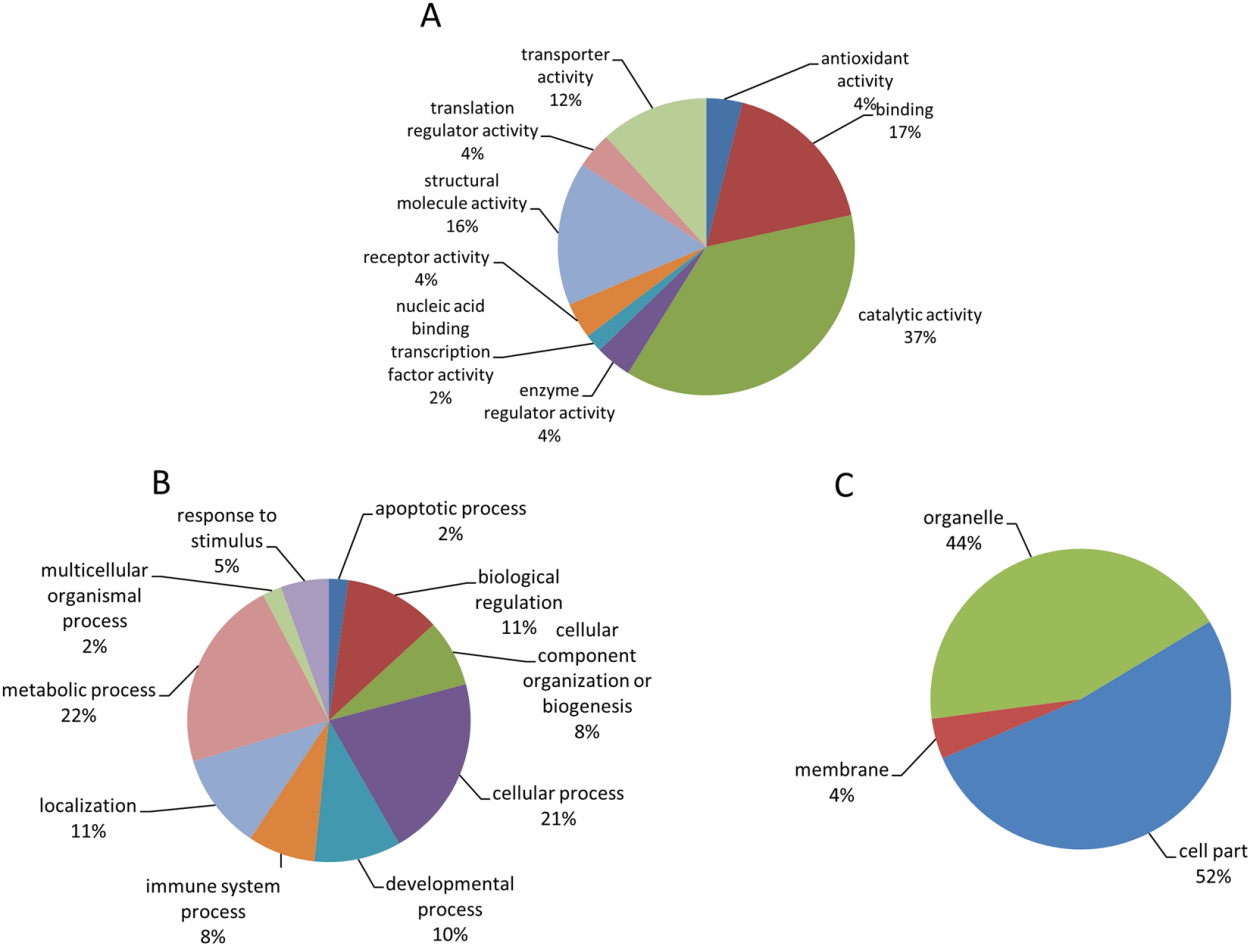
Pie charts showing the classification of differentially expressed proteins in the SYFs of layer-type L2 strain Taiwan country chickens who underwent 36 °C acute heat stress for 4 h and recovery for 0, 2, and 6 h, stratified by molecular functions (**A**), biological processes (**B**), and cellular component (**C**).

The 2D-DIGE analysis identified more than 1500 protein spots in the SYFs, with 142 protein spots differentially affected after acute heat stress (≥1.3 fold; P < 0.05; Fig. 5). Protein identification analysis showed that the differential protein spots represented 93 distinct proteins (Supplementary Table S2). Most of the proteins were localized to the cell (52%) and organelles (44%; Fig. 6), and are involved in metabolic processes (22%), cellular processes (21%), biological regulation (11%), and localization (11%). In molecular function, the differentially expressed proteins mainly participate in catalytic activity (37%), binding (17%), structural molecule activity (16%), and transporter activity (12%; Fig. 6), and are involved in apoptosis, gap junctions, glycolysis/gluconeogenesis, oxidative phosphorylation, metabolic processes, progesterone-mediated oocyte maturation, and cytoskeleton remodeling (Table 1).

**Table 1:** Pathways that involve differentially expressed proteins in SYFs of layer-type L2 strain Taiwan country chickens after acute heat stress.

KEGG pathway	Related proteins and their expression in chicken SYF after heat stress^#^
Apoptosis	PIK3CG (NS,↑,↑); TUBA1C (NS,↑,↑); ACTG1 (NS,↑,↑)
Carbon metabolism	ENO1 (NS,↑,↑); GAPDH (NS,↑,↑); TPI1 (NS,↑,↑)
Gap junction	TUBA1C (NS,↑,↑); TUBB2A (NS,↑,↑); TUBB2B (NS,↑,↑); TUBB4B (NS,↑,↑)
Glycolysis/Gluconeogenesis	ENO1 (NS,↑,↑); GAPDH (NS,↑,↑); TPI1 (NS,↑,↑)
Inositol phosphate metabolism	TPI1 (NS,↑,↑); PIK3CG (NS,↑,↑)
Oxidative phosphorylation	ATP5A1W (NS,↑,↑); ATP5A1W (NS,↑,↑); ATP5B (↓,↑,↑); NDUFA10 (NS,↑,↑)
Metabolic pathways	ENO1 (NS,↑,↑); GAPDH (NS,↑,↑); TPI1 (NS,↑,↑); ATP5A1W (NS,↑,↑); ATP5A1W (NS,↑,↑); ATP5B (↓,↑,↑); NDUFA10 (NS,↑,↑)
Peroxisome	SOD2 (NS,↑,↑); PRDX1 (NS,↑,↑)
Progesterone-mediated oocyte maturation	PIK3CG (NS,↑,↑)
Proteasome	PSMA5 (NS,↑,↑)
Protein processing in endoplasmic reticulum	CKAP4 (↑,↑,↑); ERP29 (NS,↑,NS);HSP70 (NS,↑,↑); P4HB (NS,↑,↑); TXNDC5 (NS,↑,↑)
Regulation of actin cytoskeleton	PIK3CG (NS,↑,↑); ACTG1 (NS,↑,↑); CTC-554D6.1 (NS,↑,↑)
Ribosome	RPLP0 (NS,↑,NS)
RNA degradation	ENO1 (NS,↑,↑); HSPD1 (↑,↑,↑); HSPA9 (NS,↑,↑)
Tight junction	ACTG1 (NS,↑,↑)
Specific signaling pathway	
mTOR signaling pathway	PIK3CG (NS,↑,↑)
Toll-like receptor signaling pathway	PIK3CG (NS,↑,↑)
Wnt signaling pathway	CTC-554D6.1 (NS,↑,↑)

^#^(H4R0/CTL, H4R2/CTL, H4R6/CTL), ↑, with an increase after heat stress; ↓, with a decrease after heat stress.

### Validation of protein expression in SYFs of layer-type L2 strain TCCs

**Figure 7:**
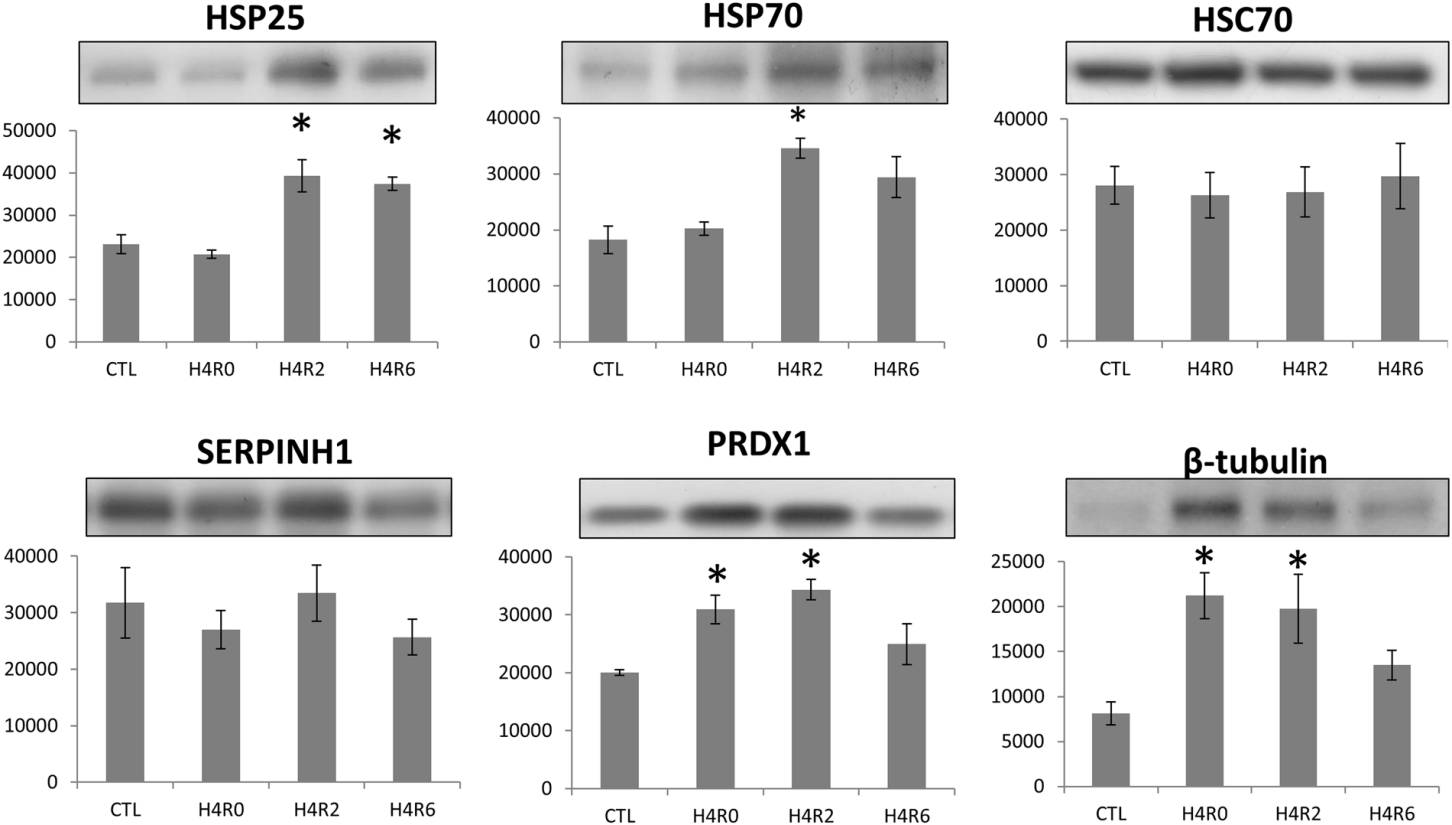
Western blot analysis of differential expressed proteins in the SYFs of layer-type L2 strain Taiwan country chickens after acute heat stress. The asterisk indicate the values differ significantly between the heat-stressed and control groups (P < 0.05). The blot profiles are cropped to show one replicate of western blot analysis. The raw profiles are showed in supplementary Fig. S2.

In the validation by Western blot analysis, the expressions of HSP25, HSP70, PRDX1, and β-tubulin after acute heat stress coincided with the observations in 2D-DIGE analysis. However, HSC70 and SERPINH1 expressions were not affected by acute heat stress (Fig. 7).

## Discussion

SYFs reside in the prehierarchical stage and represent a critical pool of follicles for selection into the hierarchy that is tightly associated with laying performance^16^. Consistent with the results of our previous studies^21–25^, the respiratory rate and rectal temperature markedly increased after the initiation of acute heat stress (Fig. 1). In contrast to broilers^26^, however, a sluggish response of body temperature change after acute heat stress was observed in TCC, suggesting their better heat tolerance. After acute heat stress, 176 transcripts and 93 distinct proteins were significantly altered in terms of their abundances (Supplementary Tables S1 and S2). In the microarray analysis, the 2-fold variations of mRNAs with different probes of the same features were observed in the present study (Supplementary Table S1). In the proteomic analysis, the features of abundance variations with over 1.3-fold changes in different spots of the same protein were also observed (Fig. 5; Supplementary Table S2). These results may be attributed to the post transcriptional/translational modifications or by truncated fragments from a protein due to degradation. However, most of these different spots on SDS-PAGE of the same identified protein behaved with consistent changes to acute heat stress. Most of the differential protein expressions were altered within 2 h of recovery, mainly involved in the biological processes of apoptosis, biological regulation, cellular component organization, biogenesis, cellular processes, developmental processes, immune system processes, localization, metabolic processes, multicellular organismal processes, and responses to stimuli. Based on the bioinformatics analysis, the differential gene expressions related to the responses to stimuli and reproduction were validated through qRT-PCR or Western blot analyses. Most of the results, however, were not consistent with those of microarray and 2D-DIGE analyses, possibly due to the probe or primer sequence design. Besides, a lower mRNA level might also reflect the low correlation between microarray and qRT-PCR analyses^27^. In 2D-DIGE and Western blot analyses, the inconsistent results may be attributed to the different isoforms, subunits, and motifs recognized by the antibodies, whereas Mass spectra truly reflects fragment identities. Moreover, the different protein sample preparations may also cause the inconsistency. Despite the discrepancies, the following discussion is mainly focused on the validated results and differentially expressed genes and proteins related to heat stress response, oxidative stress and egg production.

Excessive reactive oxygen species (ROS) cause cell oxidative stress and injuries, such as DNA and protein damages, and may lead to cell death^28^. Heat stress increases ROS generation in follicles^29^. In this study, the transcript levels of Sesn2, a ROS-responsive gene acting on antioxidative defense through p53-dependent regulation of autophagy and cell viability^30^, and PRDX4, which operates at thioredoxin for redox regulation, were upregulated within 2 h of recovery after heat stress (Supplementary Table S1). Besides, SOD2, PRDX1, and PRDX3 protein abundance also increased in response to acute heat stress (Supplementary Table S2). Both mRNA and protein levels of PRDX4 were increased by H_2_O_2_ in a dose-dependent manner and the changes were closely related to cell proliferation^31^. Superoxide dismutases (SODs) functioning in ROS clearance are recognized for their regulations in in cell growth, metabolism, and oxidative stress responses^32^. Accordingly, acute heat stress may elicit oxidative stress and thereby activates cellular antioxidative processes and stress-responsive factors to protect the follicle cells from death.

Heat shock proteins (HSPs) as chaperones to relieve endoplasmic reticulum stress can function to protect cells from death^33^ in response to various stimuli such as heat stress, oxidative stress, chemical damage, and ultraviolet exposure^34–39^. The transcript levels of Hsp25, Hsp90aa1, Hsp90b1, and Hspa5 were significantly upregulated in the SYFs after acute heat stress while Hsp90aa1 and Hspa8 were downregulated after 2 h of recovery (Supplementary Table S1). In proteomic analysis, HSP70, TRAP1, HSPA9, HSPD1, and HSP25 abundance were upregulated after acute heat stress but HSP108 and SERPINH1 were downregulated first and upregulated after 2 h of recovery (Supplementary Table S2). In consistence with our previous reports^21–24^, upregulation of HSP expressions of ovarian SYFs reproductive tissues after acute heat stress may suggest a protective mechanism against stress. HSP25 has been shown with differential expressions in the brain, liver, and leg muscle of chickens after acute heat exposure^40^ and the present results also demonstrated highly differential changes of HSP25 expression in SYFs in consistent with our previous observations in testes^23^. HSP27, a homolog of HSP25 in chicken, specifically acts on cytosolic cytochrome c to attenuate caspase activation and the downstream apoptotic pathway^41^. Regulation of HSP27 in cell apoptosis have been observed in mouse oocytes^41^ and in the granulosa cells of the secondary, tertiary, and cystic follicles in cows^42^. Accordingly, upregulation of HSP25 might prevent the apoptosis of SYFs and follicular atresia induced by acute heat stress in the layer chickens.

In qRT-PCR analysis, the levels (H4R0 and H4R2) of Hsd17b1 decreased after acute heat stress (Fig. 4), which may implicate impaired production of sex steroids^43^ due to the dysfunction of ovarian metabolic activities^42^ and cytoskeletons (β-tubulin) in maintaining cell shape integrity^44,45^. Different isoforms of VTG proteins of SYFs were upregulated after heat stress (Supplementary Table S2). During the laying stage, hens have to synthesize massive constituents of egg yolk, such as lipids, apolipoproteins (APOs), and VTG in the liver^46^, Thus it cannot exclude the possibility that the proteins are blood borne from hepatic origin despite that the samples were rinsed several times. Further studies are required to confirm the origin of APOB and VTG of the SYF tissues and their changes in response to acute heat stress.

Genomic regulation analysis demonstrated that only the differential changes of APOB, HSP25, PRDX4, and SERPINH1 were consistent at both the transcript and proteins levels (Supplementary Tables S1 and S2). Previous studies have shown a quite varied correlationship between responsive transcript and corresponding protein levels^47,48^. Proteins are the major executors to regulate biological functions more directly than do mRNAs^48^. In addition, the transcriptional elements are not always translated into functional objects. Some conjectures exist regarding this phenomenon in the present study. Firstly, the expression of some messengers might not be sufficient for the translation threshold of mRNAs of the SYFs after acute heat stress. By contrast, the slight but essential expression of mRNAs might be ignored by the statistical condition (two-fold changes in expression). Finally, some novel undefined mechanisms might exist that would antagonize or provide a feedback for the differentially expressed mRNAs, suggesting an inadmissibility in the predicting protein levels from their transcripts. Furthermore, the inconsistency of mRNA and protein expressions may be attributed to the differential effects of acute heat stress on the translation and transcription machinery in SYFs. The present study reported the global regulation of transcripts and proteins by acute heat stress in attempt to monitor intact SYFs response instead of specifying on a certain cell type. A functional genomic regulation of specific SYF cells in response to acute heat stress requires further investigations.

## Conclusion

This study demonstrates the transcriptomic and proteomic regulations of SYFs in layer-type L2 strain TCCs in response to acute heat stress. A total of 176 genes and 93 distinct proteins were differentially expressed after acute heat stress. Genes related to apoptotic and antioxidative activities, such as the HSP and PRDX families, were upregulated.

## Materials and Methods

### Experimental animals and managements

Twelve 30-week-old layer-type L2 strain TCC hens originally selected for egg production from National Chung Hsing University^49,50^ were used. The experimental protocols, and care and use of all animals were complied with the guidelines and approved by the Institutional Animal Care and Use Committee of National Chung Hsing University (Taichung, Taiwan; IACUC No. 102-06). For more than two weeks, hens were maintained in a climate chamber for adaptation under conditions of a light:dark photoperiod of 14:10 h at 25°C and 55% relative humidity (RH) before acute heat stress treatment. Feed and water were provided *ad libitum*.

### Condition for acute heat stress and sample collection

After the adaptation period, nine of the 12 hens were subjected to acute heat stress at 36 °C and 55% RH for 4 h, and the other three hens were maintained at 25°C and 55% RH as a control (CTL) group. Heat-stressed hens were allowed to recover for 0, 2, and 6 h after heat stress (defined as the H4R0, H4R2, and H4R6 groups, respectively) at 25°C and 55% RH. The respiratory rate and body temperature were recorded before and during heat stress and recovery periods. The respiratory rate was measured by counting the panting breaths and the body temperature was obtained by introducing an alcohol thermometer into the cloaca of the chickens. Hens were euthanized at each time point for necropsy, and SYF samples were collected and stored at −80°C for further analyses.

### Gene expression analysis in response to acute heat stress by microarray analysis

The chicken 44 K oligo microarray V2 (Design ID: 026441, Agilent Technologies, Santa Clara, CA, USA) was used to determine the differential gene expressions between the CTL and acute heat-stressed groups, as described in our previous study^24^. SYF tissues from each animal sample were used for RNA isolation and further analysis independently. For reverse transcription, second strand complementary (c)DNA was synthesized from 1 μg of total RNA using a Quick-Amp Labeling kit (Agilent Technologies). Microarrays were scanned using an Agilent microarray scanner (Agilent Technologies) at 535 nm for Cy3. Scanned images were analyzed using Feature Extraction 10.5.1.1 software (Agilent Technologies). Results of the microarray analysis were filtered from the features when flags were present or were marginal in at least one of the four (CTL, H4R0, H4R2, and H4R6) groups. For comparison between the CTL and heat-stressed groups, gene expressions with more than two-fold changes (up or down) were considered as significantly different.

### Validation of gene expression through quantitative real-time polymerase chain reaction

The transcript levels of eight genes, including heat shock protein 25 (Hsp25); heat shock protein 90 kDa alpha, class A member 1 (Hsp90aa1); cold-inducible RNA-binding protein (Cirbp); apolipoprotein B (ApoB); peroxiredoxin 4 (Prdx4); cytochrome P450, family 19, subfamily A, polypeptide 1 (Cyp19a1); hydroxysteroid 17-beta dehydrogenase 1 (Hsd17b1); and heat shock protein 47 (Serpinh1) were selected after bioinformatics analysis for validation through a quantitative real-time polymerase chain reaction (qRT-PCR) analysis. The samples used in the microarray analysis were also used for validation. Primers for the qRT-PCR and amplicon sizes are shown in Table 2. The transcript level of each of the target gene was normalized to 18s rRNA and calculated as a ratio relative to the control^23^. For consistency with the microarray analysis, the cutoff value for the differentially expressed genes was set to a two-fold or higher change.

**Table 2:** Primers and product size of genes used for validation using quantitative real-time polymerase chain reaction.

Gene symbol^#^	GenBank accession number	Forward (F) primers (5′-3′)	Product size (base pair)
		Reverse (R) primers (5′-3′)	
Hsp25	NM_001010842	F: CCGTCTTCTGCTGAGAGGAGTG	117
		R: ACCGTTGTTCCGTCCCATCAC	
Hsp90aa1	NM_001109785	F: GGTGTTGGTTCCTACTCTGCTTAC	76
		R: ACTGCTCATCATCATTGTGCTTGG	
Cirbp	NM_001031347	F: GCCTGGGTACAAATTGGAAG	72
		R: GCAGGTTGAACATACAAGCAAG	
ApoB	NM_001044633	F: TTCCAGCTTCCACGTATCCC	181
		R: ATTTGGACGTTGCTTGAGCTG	
Prdx4	BX935739	F: ACATGCACTTAGGGGCCTTTT	90
		R: TCCACTGATCTCCCCACAGG	
Cyp19a1	NM_001001761	F: AGACGGTGCAGAGTACAGAC	92
		R: AGGCTGCCTTTCTATTGGGTG	
Hsd17b1	NM_204837	F: CTCGGAGCAGGCCATGAGAG	70
		R: GAAGGCCTGAATGGTGCGT	
Serpinh1	NM_205291	F: AGCGCCCTGAAATCCATCAA	171
		R: GAAGCCACGGTTATCCACCA	
18s rRNA	KT445934.2	F: GGGATGCAGATCTTCGTGAAA	117
		R: CAAAAGCTTGTGTCGAGGGC	

^#^Abbreviations: Hsp25, heat shock protein 25 gene; Hsp90aa1, heat shock protein 90 alpha gene; Cirbp, cold-inducible RNA-binding protein gene; ApoB, apolipoprotein B gene; Prdx4, peroxiredoxin 1 gene; Cyp19a1, cytochrome P450, family 19, subfamily A, polypeptide 1 gene; Hsd17b1, hydroxysteroid 17-beta dehydrogenase 1 gene; Serpinh1, heat shock protein 47 gene.

### Protein expression analysis in the SYFs of layer-type L2 strain TCCs after acute heat stress by using two-dimensional difference gel electrophoresis

#### Sample preparation for two-dimensional difference gel electrophoresis analysis

All SYF samples were sliced and washed in sucrose buffer (10 mmol/L Tris/HCl, 250 mmol sucrose, pH 7) and centrifuged at 4°C (400 × g; three times for 5 min each) to analyze the global proteomic regulations of whole SYF tissue. The supernatant was collected and lysed in O’Farrell’s lysis buffer (9.5 M urea, 2% NP-40, 2% v/v Ampholyte 3-10, and 65 mM dithiothreitol). The samples were further sonicated (40 w; six times for 10 s) to dissolute proteins from SYFs. The total protein quantity was determined using the Bradford Protein Assay (Bio-Rad, Hercules, USA). An equal amount of proteins from each of the three individuals of the same group (CTL, H4R0, H4R2, and H4R6 groups) was pooled and dried using speed vacuum (CVE- 200D; EYELA, Tokyo, Japan). All samples were dissolved in 2-DE lysis buffer (4% wt/vol 3-[(3-Cholamidopropyl) dimethylammonio]-1-propanesulfonate, 7 M urea, 2 M thiourea, 10 mM Tris-HCl, pH 8.3, 1 mM [Octylphenoxy] polyethoxyethanol) for two-dimensional difference gel electrophoresis (2D-DIGE) analysis.

#### 2D-DIGE and image analysis

The procedures for 2D-DIGE analysis followed the methods of Wang *et al*.^23^. In brief, 100 mg of proteins were labeled with 250 pmol of either Cyanine dye 3 (Cy3) or Cy5 for comparison on the same gel. To facilitate image matching and cross-gel statistical comparison, a pool of all samples of each treatment was labeled with Cy2 as an internal standard for all gels. The equilibrated strips with reduced SYF samples were subjected to second dimension separation through 12.5% sodium dodecyl sulfate-polyacrylamide gel electrophoresis. After electrophoresis, the gels were scanned using the Ettan DIGE Imager (GE Healthcare, Uppsala, Sweden). Protein spots on all the gels were detected, normalized, and analyzed using DeCyder 2D Differential Analysis Software (Version 7.0, GE Healthcare). Spots with an average of 1.3-fold changes in abundance, with a P value of <0.05, were considered significantly different. For spot picking, an additional aliquot (400 μg) of the SYF sample was analyzed. Gel staining, spot cleaning, and in-gel digestion of differentially expressed protein spots were performed in accordance with the procedures of Wang *et al*.^23^.

#### Protein identification

Protein identities were analyzed using a matrix-assisted laser desorption/ionization time-of-flight mass spectrometer (MALDI-TOF MS), as described by Lu *et al*.^51^. Mass spectra were processed using FlexAnalysis 2.4 software (Bruker Daltonics, Bremen, Germany). Peptide masses were searched against a comprehensive nonredundant protein sequence database (NCBInr 20130524 version with 25805290 sequences and 8915431356 residues; SwissProt 2013_05 version with 540052 and 191770152 residues) employing BioTools 3.0 software (Bruker Daltonics) in combination with the MASCOT program^22^. Protein identities were further confirmed through MALDI TOF/TOF MS/MS analysis by using a Bruker UltraFlex III MALDI-TOF/TOF MS (Bruker Daltonics) equipped with a delayed extraction ion source. The metastable ions generated by laser-induced decomposition (LID) in the laser-induced forward transfer (LIFT) mode (Bruker Daltonics) was analyzed. The MS/MS spectra were searched against the NCBInr database (NCBInr 20130524 version with 25805290 sequences and 8915431356 residues; SwissProt 2013_05 version with 540052 and 191770152 residues) using Bio-Tools 3.0 software (Bruker Daltonics) in combination with the MASCOT program.

### Western blot analysis of differential expressed proteins in SYFs of a layer-type L2 strain TCCs after acute heat stress

The procedure for western blot analysis was modified from the methods of Wang *et al*.^21^. Soluble proteins from chicken SYFs were lysed in a sample buffer and separated using a TGX Stain-Free FastCast Acrylamide Starter Kit (161-0184, Bio-Rad, Hercules, CA, USA). The proteins were then transferred to an Immun-Blot® Low Fluorescence PVDF membrane (Bio-Rad) and blocked in 20 mM Tris-HCl (pH 7.4), 0.5 M NaCl, and 0.05% Tween 20 (TTBS) containing 3% gelatin for 1 h. The membranes were immunoblotted with a 1:1000–diluted anti-heat shock protein 70 (HSP70) rabbit polyclonal antibody (SPA-812; EnZo Life Science, Farmingade, NY, USA), 1:1000–diluted anti-heat shock cognate 70 (HSC70) rat polyclonal antibody (SPA-815; EnZo Life Science), 1:400–diluted anti-heat shock protein 25 (HSP25) mouse polyclonal antibody (sc-51956; Santa Cruz Biotechnology, Inc., Dallas, TX, USA), 1:200–diluted anti-heat shock protein 47 (HSP47) rabbit polyclonal antibody (LC-B2151; LifeSpan BioScience, Seattle, WA, USA), 1:1000–diluted anti-peroxiredoxin-1 (PRDX1) rabbit polyclonal antibody (ARP48454_P050; Aviva Systems Biology, San Diego, CA, USA), or 1:200–diluted anti-β-tubulin rabbit polyclonal antibody (sc-9104; Santa Cruz Biotechnology, Inc.) for 1 h. After washing three times in TTBS, the membranes were incubated with 1:5000–diluted goat anti-mouse, goat anti-rabbit, or goat anti-rat immunoglobulin G (IgG) conjugated with alkaline phosphatase (Sigma, St. Louis, MA, USA) for matching the primary antibody used. The density of immunoblotted protein bands was determined using ImageJ software (Version 1.51 f, LSU Health Sciences Center, New Orleans, LA, USA). The expression levels of proteins were obtained by normalizing the density of protein bands to the total protein by using the Image Lab software (Version 5.2.1, Bio-Rad).

### Bioinformatics analysis

The differential mRNAs and proteins among treatments were annotated for their subcellular distribution, biological processes, and molecular functions by using the Gene Ontology database (http://www.geneontology.org/).

### Statistical analysis

The comparison of physiological parameters during acute heat stress between the CTL and heat-stressed hens and the relative values of proteins on 2D-DIGE gels or on membranes of western blot analysis were analyzed through the Student t test by using the Statistical Analysis System Software^52^. Multiples of changes in microarray and qRT-PCR analyses were presented as the arithmetic mean of three replicates from every individual of each group.

### Data availability statement

All data are fully available without restriction. All relevant data are within the paper and its Supporting Information files. The dataset of microarray analysis has been submitted to Gene Expression Omnibus in the National Center for Biotechnology Information under the accession number of GSE99108.

### Ethical approval and informed consent Approval

The care and use of all animals in the study were complied with the guidelines and was approved by the Institutional Animal Care and Use Committee of National Chung Hsing University (Taichung, Taiwan; IACUC No. 102-06). The approving body has been identified in the methods section.

## Electronic supplementary material

Supplementary information
