# Supplementary material for: Functional genomics study of acute heat stress response in the small yellow follicles of layer-type chickens
Source: Sci Rep. 2018 Jan 22;8:1320. doi: 10.1038/s41598-017-18335-5 (PMC5778056; doi:10.1038/s41598-017-18335-5)
Supplement: Supplementary file 1 — Supplementary information [file 41598_2017_18335_MOESM1_ESM.pdf]

Functional genomics study of acute heat stress response in the small yellow follicles of layer-type chickens

Chuen-Yu Cheng[1], Wei-Lin Tu[1], Chao-Jung Chen[2,3], Hong-Lin Chan[4,5], Chih-Feng Chen[1,6,7], Hsin-Hsin Chen[1], Pin-Chi Tang[1,2], Yen-Pai Lee[1], Shuen-Ei Chen[1,6,7,*] and San-Yuan Huang[1,6,7,8,*]

Supplementary information

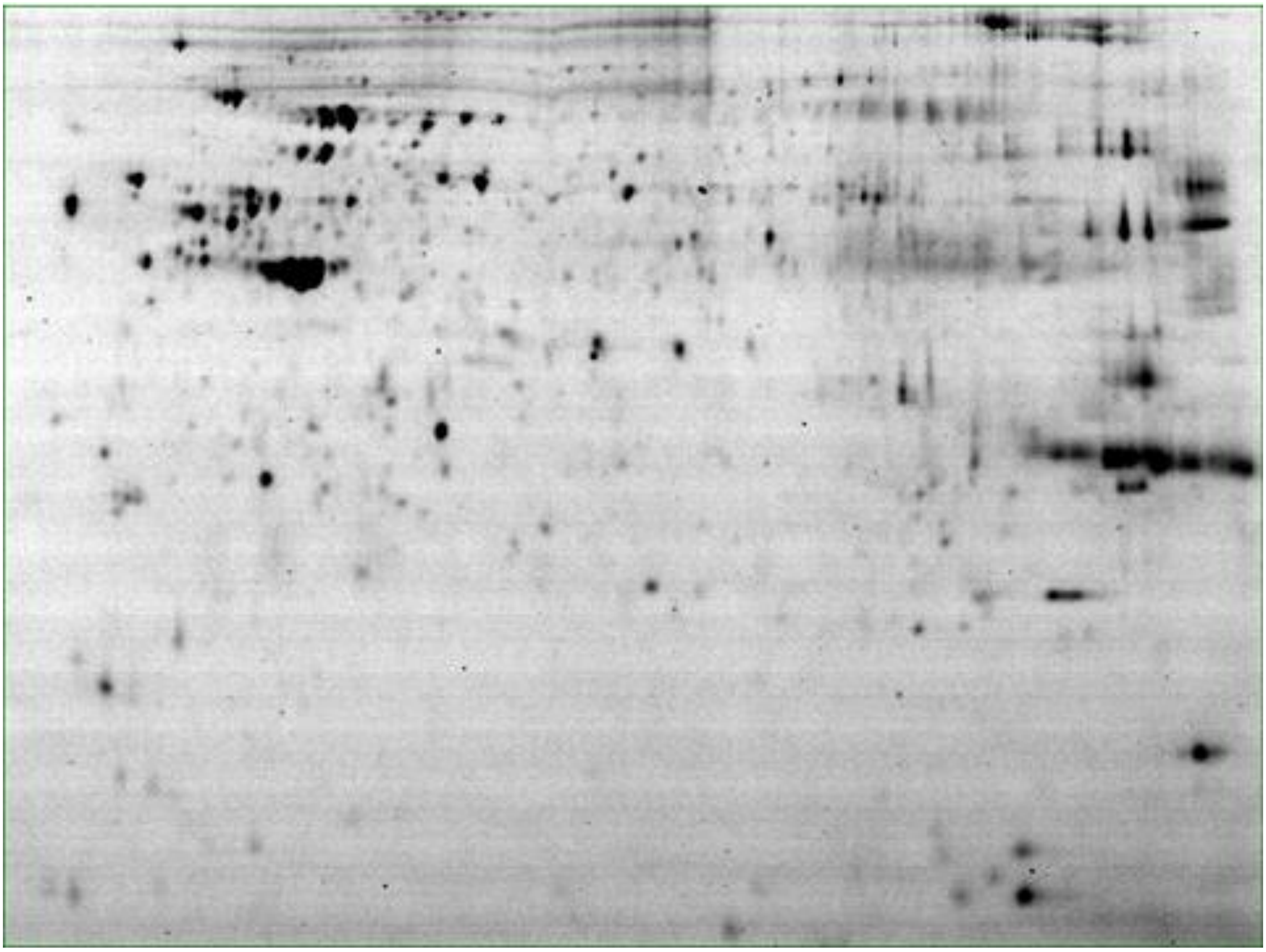

Supplementary Fig. S1. The two dimensional difference gel electrophoresis raw patterns of SYFs of L2 strain Taiwan country chicken.

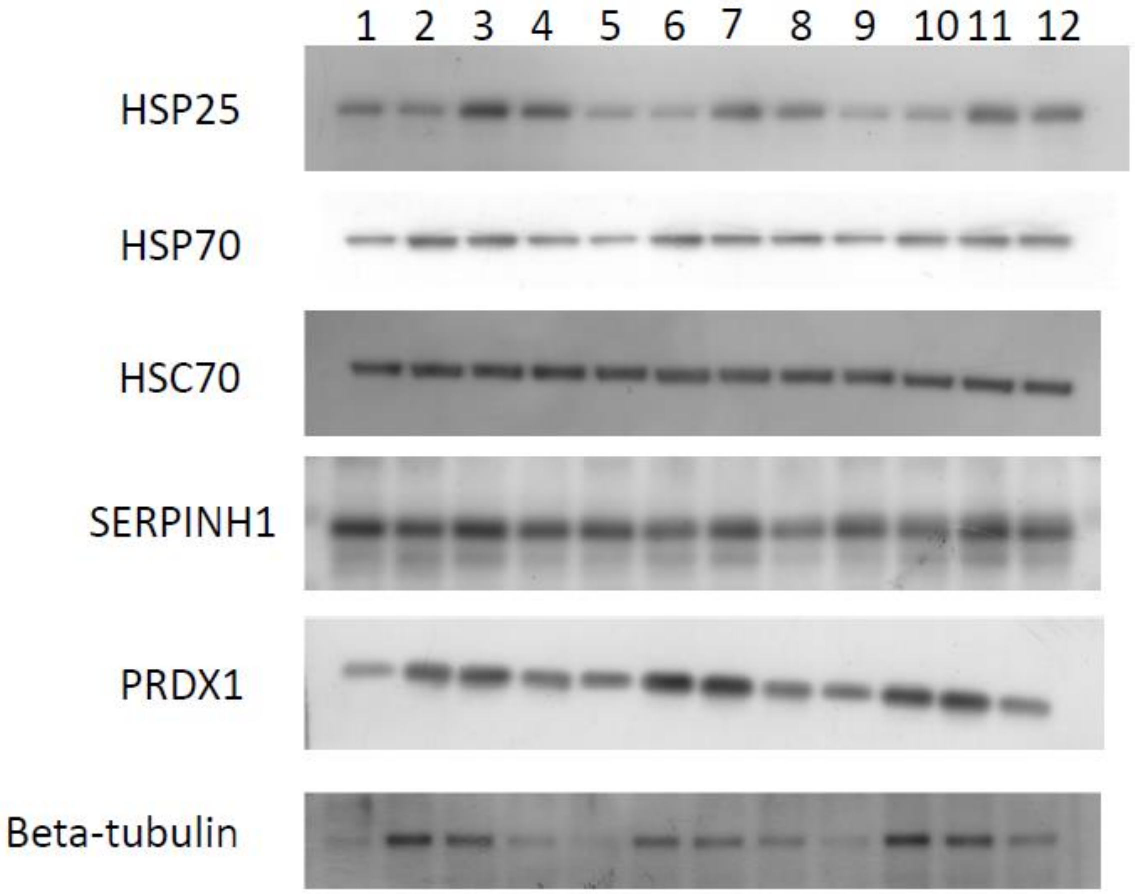

Supplementary Fig. S2. The raw patterns of western blot analysis. HSP25, heat shock protein 25; HSP70, heat shock protein 70; HSC70, heat shock cognate 70; SERPINH1, heat shock protein 47; PRDX1, peroxiredoxin-1. Column 1, 5, and 9, control group; Column 2, 6, and 10, acute heat stressed and without recovery period group; Column 3, 7, and 11, acute heat stressed with 2 h recovery period group; Column 4, 8, and 12, acute heat stressed with 6 h recovery period group.

Supplementary Table S1. The differentially expressed mRNAs in SYFs of layer-type L2 strain Taiwan country chickens after acute heat stress#.

Probe Name	H4R0/CTL	H4R2/CTL	H4R6/CTL	Gene symbol	GenBank accession	UniGene ID	Description
A_87_P052466	-1.311	-1.160	-2.101	AATF	NM_001030724	Gga.7407	Gallus gallus apoptosis antagonizing transcription factor (AATF), mRNA [NM_001030724]
A_87_P132523	2.057	2.370	2.151	ACHE	AJ698861	Gga.52373	Gallus gallus partial mRNA for acetylcholinesterase (ACHE gene). [AJ698861]
A_87_P056391	1.382	2.109	1.381	ACSL5	NM_001031237	Gga.7847	Gallus gallus acyl-CoA synthetase long-chain family member 5 (ACSL5), mRNA [NM_001031237]
A_87_P027203	1.316	-2.014	1.420	ACVR1C	BU428138	Gga.31405	activin A receptor, type IC [Source:HGNC Symbol;Acc:18123] [ENSGALT00000020491]
A_87_P024780	-1.501	-1.312	-2.128	ADAMTSL3	BX929295	Gga.55480	ADAMTS-like 3 [Source:HGNC Symbol;Acc:14633] [ENSGALT00000009854]
A_87_P099416	-1.031	-2.390	-2.673	AKD1	XM_004940311		adenylate kinase 9 [Source:HGNC Symbol;Acc:33814] [ENSGALT00000024568]
A_87_P021960	1.336	1.367	2.182	ANO1	BX935750	Gga.53861	Gallus gallus finished cDNA, clone ChEST41d3. [BX935750]
A_87_P012184	1.770	2.229	1.043	APOB	NM_001044633	Gga.4112	Gallus gallus apolipoprotein B (including Ag(x) antigen) (APOB), mRNA [NM_001044633]
A_87_P012759	2.052	-1.107	1.484	AQP12	NM_001109679	Gga.53127	Gallus gallus aquaporin 12 (AQP12), mRNA [NM_001109679]
A_87_P294402	2.350	1.247	1.429	ARIH1	NM_001030387	Gga.53210	Gallus gallus ariadne homolog, ubiquitin-conjugating enzyme E2 binding protein, 1 (Drosophila) (ARIH1), mRNA [NM_001030387]
A_87_P075601	1.933	2.670	2.474	B2M	NM_001001750	Gga.1328	Gallus gallus beta-2-microglobulin (B2M), mRNA [NM_001001750]
A_87_P120898	2.486	-1.390	1.418	BAIAP2L2	CR385417	Gga.15220	Gallus gallus finished cDNA, clone ChEST596f1. [CR385417]
A_87_P302598	1.366	2.051	1.128	C2H6ORF62	CR523630	Gga.20197	chromosome 6 open reading frame 62 [Source:HGNC Symbol;Acc:20998] [ENSGALT00000030973]
A_87_P260383	1.483	2.383	1.317	C2H8ORF84	XM_429743		somatomedin B and thrombospondin, type 1 domain containing [Source:HGNC Symbol;Acc:30362] [ENSGALT00000025208]
A_87_P060201	-2.942	-1.709	-5.853	C4	NM_001077233	Gga.16394	Gallus gallus complement 4 (C4), mRNA [NM_001077233]
A_87_P025992	-1.641	-2.170	-2.705	C5	BU463842	Gga.27675	complement component 5 [Source:HGNC Symbol;Acc:1331] [ENSGALT00000002379]
A_87_P085261	1.072	2.087	1.337	C5H14ORF45	CR405825	Gga.11956	coiled-coil domain containing 176 [Source:HGNC Symbol;Acc:19855] [ENSGALT00000032568]
A_87_P130708	-1.713	-2.058	-1.124	CA2	NM_205317	Gga.3986	Gallus gallus carbonic anhydrase II (CA2), mRNA [NM_205317]
A_87_P023735	1.989	3.287	4.033	CA5B	BX931926	Gga.53563	Uncharacterized protein [Source:UniProtKB/TrEMBL;Acc:F1N986] [ENSGALT00000009408]
A_87_P075221	2.350	3.452	4.779	CA5B	BX931926	Gga.53563	Uncharacterized protein [Source:UniProtKB/TrEMBL;Acc:F1N986] [ENSGALT00000009408]
A_87_P217153	-1.502	-1.852	-2.474	CA7	BU123298	Gga.40333	carbonic anhydrase VII [Source:HGNC Symbol;Acc:1381] [ENSGALT00000008318]
A_87_P035639	-1.273	2.040	1.266	CASD1	AJ851642	Gga.31495	Gallus gallus mRNA for hypothetical protein, clone 14m6. [AJ851642]
A_87_P072166	-1.358	-2.147	-1.758	CCDC78	BU366103	Gga.45507	Uncharacterized protein [Source:UniProtKB/TrEMBL;Acc:R4GI76] [ENSGALT00000045501]
A_87_P016539	1.946	2.003	-1.160	CDKL2	CR386352	Gga.13424	cyclin-dependent kinase-like 2 (CDC2-related kinase) [Source:HGNC Symbol;Acc:1782] [ENSGALT00000016460]

A_87_P030709	1.021	-1.413	-2.027	CFTR	NM_001105666	Gga.27462	Gallus gallus cystic fibrosis transmembrane conductance regulator (ATP-binding cassette sub-family C, member 7) (CFTR), mRNA [NM_001105666]
A_87_P009694	-2.205	1.172	1.049	CIRBP	NM_001031347	Gga.42673	Gallus gallus cold inducible RNA binding protein (CIRBP), mRNA [NM_001031347]
A_87_P054071	-2.333	1.377	1.298	CIRBP	NM_001031347	Gga.42673	Gallus gallus cold inducible RNA binding protein (CIRBP), mRNA [NM_001031347]
A_87_P013516	-1.824	-1.442	-2.368	CLUAP1	CR391360	Gga.15912	Gallus gallus finished cDNA, clone ChEST264b15. [CR391360]
A_87_P008806	-1.452	-1.661	-2.276	CNTN4	X85809	Gga.55020	Uncharacterized protein [Source:UniProtKB/TrEMBL;Acc:F1P3U6] [ENSGALT00000013444]
A_87_P134355	1.464	2.152	-1.068	COR6	NM_001031544	Gga.32089	Gallus gallus chick olfactory receptor 6 (COR6), mRNA [NM_001031544]
A_87_P135305	1.758	2.494	1.357	CREB3L3	BX929691	Gga.7247	Gallus gallus finished cDNA, clone ChEST800b2. [BX929691]
A_87_P010130	2.195	1.382	-1.292	CRELD2	NM_001199497	Gga.11562	Gallus gallus cysteine-rich with EGF-like domains 2 (CRELD2), mRNA [NM_001199497]
A_87_P009329	1.097	2.437	1.556	CSNK2B	NM_001257204	Gga.34034	Gallus gallus casein kinase 2, beta polypeptide (CSNK2B), mRNA [NM_001257204]
A_87_P057226	-1.560	-1.430	-2.582	CYP19A1	NM_001001761	Gga.859	Gallus gallus cytochrome P450, family 19, subfamily A, polypeptide 1 (CYP19A1), mRNA [NM_001001761]
A_87_P008373	-1.061	-2.086	1.330	CYTB			Cytochrome b [Source:UniProtKB/Swiss-Prot;Acc:P18946] [ENSGALT00000029080]
A_87_P173053	2.097	-1.022	1.970	CYTH4	NM_001012815	Gga.12335	Gallus gallus cytohesin 4 (CYTH4), mRNA [NM_001012815]
A_87_P112188	1.931	2.250	1.113	DDX3X	NM_001030800	Gga.12607	Gallus gallus DEAD (Asp-Glu-Ala-Asp) box polypeptide 3, X-linked (DDX3X), mRNA [NM_001030800]
A_87_P297388	1.890	2.077	1.187	DDX3X	NM_001030800	Gga.12607	Gallus gallus DEAD (Asp-Glu-Ala-Asp) box polypeptide 3, X-linked (DDX3X), mRNA [NM_001030800]
A_87_P024343	-1.798	1.174	-2.696	DHRS4	NM_001277125	Gga.35194	Gallus gallus dehydrogenase/reductase (SDR family) member 4 (DHRS4), mRNA [NM_001277125]
A_87_P083476	-1.595	-2.581	-2.880	DRD4	NM_001142849	Gga.34426	Gallus gallus dopamine receptor D4 (DRD4), mRNA [NM_001142849]
A_87_P080681	-1.069	-2.035	1.046	DRGX	AB736185		Uncharacterized protein [Source:UniProtKB/TrEMBL;Acc:R4GG90] [ENSGALT00000046207]
A_87_P262338	1.004	-1.051	2.606	DSTN	NM_205528	Gga.4895	Gallus gallus destrin (actin depolymerizing factor) (DSTN), mRNA [NM_205528]
A_87_P032542	1.189	-2.770	1.691	EPB42	BU226370	Gga.44220	erythrocyte membrane protein band 4.2 [Source:HGNC Symbol;Acc:3381] [ENSGALT00000007669]
A_87_P065901	2.035	-1.107	1.554	EPHA8	XM_003642540		EPH receptor A8 [Source:HGNC Symbol;Acc:3391] [ENSGALT00000007554]
A_87_P053876	-1.633	-2.430	-1.957	F13A1	NM_204685	Gga.226	Gallus gallus coagulation factor XIII, A1 polypeptide (F13A1), mRNA [NM_204685]
A_87_P127128	-1.436	-2.276	-1.803	F13A1	CR407328	Gga.226	Gallus gallus coagulation factor XIII, A1 polypeptide (F13A1), mRNA. [Source:RefSeq mRNA;Acc:NM_204685] [ENSGALT00000037229]
A_87_P065516	1.090	-2.723	-1.492	FAM132A	BU414164	Gga.16710	family with sequence similarity 132, member A [Source:HGNC Symbol;Acc:32308] [ENSGALT00000002876]
A_87_P259653	-1.065	-2.132	-1.630	FAM132A	BU464906	Gga.51101	family with sequence similarity 132, member A [Source:HGNC Symbol;Acc:32308] [ENSGALT00000002876]
A_87_P013655	-1.456	-1.369	-2.550	FAM150A	CR391187	Gga.37597	Gallus gallus finished cDNA, clone ChEST199k16. [CR391187]
A_87_P033146	-2.501	1.082	-1.660	FAM168A	BU212749	Gga.17485	603952896F1 CSEQCHN03 Gallus gallus cDNA clone ChEST915p23 5', mRNA sequence [BU212749]
A_87_P078941	-1.377	-1.493	-2.011	FAM69A	BU474097	Gga.31895	family with sequence similarity 69, member A [Source:HGNC Symbol;Acc:32213] [ENSGALT00000009517]

A_87_P277438	-1.442	-1.696	-2.331	FBXO42	BX935690	Gga.53594	F-box protein 42 [Source:HGNC Symbol;Acc:29249] [ENSGALT00000005919]
A_87_P009222	1.841	2.196	1.159	FGF4	NM_001031546	Gga.32086	Gallus gallus fibroblast growth factor 4 (FGF4), mRNA [NM_001031546]
A_87_P096701	1.201	2.033	-1.266	FGFR3	NM_205509	Gga.14066	Gallus gallus fibroblast growth factor receptor 3 (FGFR3), mRNA [NM_205509]
A_87_P076271	1.451	-2.821	2.021	FSD2	XM_004943818		fibronectin type III and SPRY domain containing 2 [Source:HGNC Symbol;Acc:18024] [ENSGALT00000009646]
A_87_P202863	2.666	-2.430	2.617	FUT9	NM_001079502	Gga.29666	Gallus gallus fucosyltransferase 9 (alpha (1,3) fucosyltransferase) (FUT9), mRNA [NM_001079502]
A_87_P058991	-2.533	-1.855	1.018	GAL12	NM_001001607	Gga.6568	Gallus gallus beta-defensin 12 (GAL12), mRNA [NM_001001607]
A_87_P037753	-1.989	-2.549	-3.449	GATM	NM_204745	Gga.1167	Gallus gallus glycine amidinotransferase (L-arginine:glycine amidinotransferase) (GATM), mRNA [NM_204745]
A_87_P018823	1.139	2.033	1.872	GCFC1	CR338813	Gga.14840	PAX3 and PAX7 binding protein 1 [Source:HGNC Symbol;Acc:13579] [ENSGALT00000025680]
A_87_P038009	2.417	-1.331	1.911	GLI2	NM_001271901	Gga.4969	Gallus gallus GLI family zinc finger 2 (GLI2), transcript variant 1, mRNA [NM_001271901]
A_87_P010784	1.865	2.942	1.956	GREM1	NM_204978	Gga.2961	Gallus gallus gremlin 1 (GREM1), mRNA [NM_204978]
A_87_P245603	-1.208	-2.019	1.079	GRM3	XM_416842		glutamate receptor, metabotropic 3 [Source:HGNC Symbol;Acc:4595] [ENSGALT00000010624]
A_87_P009177	-1.227	-2.372	-1.066	GUCA1B	NM_001007881	Gga.624	Gallus gallus guanylate cyclase activator 1B (retina) (GUCA1B), mRNA [NM_001007881]
A_87_P063676	-1.159	-2.217	1.089	GUCA1B			Gallus gallus guanylate cyclase activator 1B (retina) (GUCA1B), mRNA. [Source:RefSeq mRNA;Acc:NM_001007881] [ENSGALT00000035143]
A_87_P112253	1.537	2.488	1.299	GYS1	AB090806	Gga.5179	Gallus gallus GS mRNA for glycogen synthase, partial cds. [AB090806]
A_87_P011151	2.005	-1.026	1.360	HCFC2	CR524095	Gga.9676	Gallus gallus finished cDNA, clone ChEST677h17. [CR524095]
A_87_P021517	-1.125	2.617	8.681	HDAC11	NM_001277141	Gga.11485	Gallus gallus histone deacetylase 11 (HDAC11), mRNA [NM_001277141]
A_87_P155283	-1.059	-2.322	1.431	HDGF	BU427752	Gga.17585	603959059F1 CSEQRBN09 Gallus gallus cDNA clone ChEST931e16 5', mRNA sequence [BU427752]
A_87_P077171	1.536	2.085	1.397	HES6	BX935736	Gga.2703	Gallus gallus finished cDNA, clone ChEST20g4. [BX935736]
A_87_P009322	-2.063	-1.236	-1.119	HMGCS1	NM_205411	Gga.4388	Gallus gallus 3-hydroxy-3-methylglutaryl-CoA synthase 1 (soluble) (HMGCS1), mRNA [NM_205411]
A_87_P024637	-1.215	-1.677	-2.207	HNMT	NM_001277873	Gga.10712	Gallus gallus histamine N-methyltransferase (HNMT), transcript variant 1, mRNA [NM_001277873]
A_87_P050016	-2.063	-1.610	-1.855	HSD17B1	NM_204837	Gga.384	Gallus gallus hydroxysteroid (17-beta) dehydrogenase 1 (HSD17B1), mRNA [NM_204837]
A_87_P005649	-1.529	-2.016	-1.636	HSD17B2	XM_414168		hydroxysteroid (17-beta) dehydrogenase 2 [Source:HGNC Symbol;Acc:5211] [ENSGALT00000008780]
A_87_P052071	7.110	4.033	1.525	HSP25	NM_001010842	Gga.985	Gallus gallus heat shock protein 25 (HSP25), mRNA [NM_001010842]
A_87_P293458	8.085	4.360	1.559	HSP25	NM_001010842	Gga.985	Gallus gallus heat shock protein 25 (HSP25), mRNA [NM_001010842]
A_87_P057951	1.506	-2.873	-1.701	HSP90AA1	NM_001109785	Gga.6482	Gallus gallus heat shock protein 90kDa alpha (cytosolic), class A member 1 (HSP90AA1), mRNA [NM_001109785]

A_87_P085771	2.030	-2.706	-1.645	HSP90AA1	NM_001109785	Gga.6482	Gallus gallus heat shock protein 90kDa alpha (cytosolic), class A member 1 (HSP90AA1), mRNA [NM_001109785]
A_87_P253769	2.421	-2.769	-1.748	HSP90AA1	NM_001109785	Gga.6482	Gallus gallus heat shock protein 90kDa alpha (cytosolic), class A member 1 (HSP90AA1), mRNA [NM_001109785]
A_87_P009402	2.312	1.155	-1.697	HSP90B1	NM_204289	Gga.4724	Gallus gallus heat shock protein 90kDa beta (Grp94), member 1 (HSP90B1), mRNA [NM_204289]
A_87_P059471	2.192	1.212	-1.808	HSP90B1	NM_204289	Gga.4724	Gallus gallus heat shock protein 90kDa beta (Grp94), member 1 (HSP90B1), mRNA [NM_204289]
A_87_P107568	2.438	1.223	-1.814	HSP90B1	NM_204289	Gga.4724	Gallus gallus heat shock protein 90kDa beta (Grp94), member 1 (HSP90B1), mRNA [NM_204289]
A_87_P295368	2.531	1.352	-1.518	HSP90B1	NM_204289	Gga.4724	Gallus gallus heat shock protein 90kDa beta (Grp94), member 1 (HSP90B1), mRNA [NM_204289]
A_87_P009374	2.023	-1.786	-1.375	HSPA5	NM_205491	Gga.4219	Gallus gallus heat shock 70kDa protein 5 (glucose-regulated protein, 78kDa) (HSPA5), mRNA [NM_205491]
A_87_P195928	2.074	-1.848	-1.416	HSPA5	NM_205491	Gga.4219	Gallus gallus heat shock 70kDa protein 5 (glucose-regulated protein, 78kDa) (HSPA5), mRNA [NM_205491]
A_87_P195043	1.717	-2.138	-1.413	HSPA8	CR386963	Gga.4555	Gallus gallus heat shock 70kDa protein 8 (HSPA8), mRNA. [Source:RefSeq mRNA;Acc:NM_205003] [ENSGALT00000010524]
A_87_P197373	1.862	-3.138	-1.668	HSPA8	NM_205003	Gga.4555	Gallus gallus heat shock 70kDa protein 8 (HSPA8), mRNA [NM_205003]
A_87_P322137	1.730	-2.694	-1.599	HSPA8	NM_205003	Gga.4555	Gallus gallus heat shock 70kDa protein 8 (HSPA8), mRNA [NM_205003]
A_87_P028311	2.974	1.590	1.629	HTATIP2	BU386490	Gga.5441	603583668F1 CSEQCHN75 Gallus gallus cDNA clone ChEST536g18 5', mRNA sequence [BU386490]
A_87_P026216	1.800	2.153	1.044	INS	BU458036	Gga.673	603368994F1 CSEQRBN19 Gallus gallus cDNA clone ChEST272l24 5', mRNA sequence [BU458036]
A_87_P081116	1.814	2.022	-1.018	ITPRIP	XM_004942269		inositol 1,4,5-trisphosphate receptor interacting protein [Source:HGNC Symbol;Acc:29370] [ENSGALT00000046035]
A_87_P037130	1.560	-2.275	-1.157	JMJD6	NM_001030703	Gga.21114	Gallus gallus jumonji domain containing 6 (JMJD6), mRNA [NM_001030703]
A_87_P126743	-1.070	-1.604	-2.199	KERA	NM_204176	Gga.4837	Gallus gallus keratocan (KERA), mRNA [NM_204176]
A_87_P198683	-1.175	-2.390	-3.219	KERA	NM_204176	Gga.4837	Gallus gallus keratocan (KERA), mRNA [NM_204176]
A_87_P107093	1.394	2.076	1.185	KIAA1467	NM_001012812	Gga.13049	Gallus gallus KIAA1467 (KIAA1467), mRNA [NM_001012812]
A_87_P152008	-2.165	1.072	-1.548	KLHDC8A	XM_004934932		kelch domain containing 8A [Source:HGNC Symbol;Acc:25573] [ENSGALT00000000967]
A_87_P021804	-2.362	-1.636	-2.424	KLHDC8B	BX936097	Gga.2732	kelch domain containing 8B [Source:HGNC Symbol;Acc:28557] [ENSGALT00000010963]
A_87_P299498	2.320	1.365	1.813	LIN7A	XM_004937594		lin-7 homolog A (C. elegans) [Source:HGNC Symbol;Acc:17787] [ENSGALT00000017803]
A_87_P176103	-2.848	1.412	-1.341	LMOD1	XM_004934867		leiomodin 1 (smooth muscle) [Source:HGNC Symbol;Acc:6647] [ENSGALT00000045637]
A_87_P023779	2.166	1.861	1.405	LNX1	BX931803	Gga.10912	Gallus gallus finished cDNA, clone ChEST790g9. [BX931803]
A_87_P077531	1.775	2.251	1.379	LOC100857766	CO771112	Gga.21517	Uncharacterized protein [Source:UniProtKB/TrEMBL;Acc:F1P116] [ENSGALT00000033325]
A_87_P061921	1.654	2.422	1.157	LOC100858410	XM_004948840		Uncharacterized protein [Source:UniProtKB/TrEMBL;Acc:F1P5I6] [ENSGALT00000003060]

A_87_P125568	-2.273	-2.176	-2.309	LOC100859660	CR391197	Gga.44840	Gallus gallus finished cDNA, clone ChEST208a13. [CR391197]
A_87_P015205	1.722	2.471	1.712	LOC100859899	CR388828	Gga.17844	Gallus gallus finished cDNA, clone ChEST693b1. [CR388828]
A_87_P078146	2.287	2.616	1.355	LOC101750121	XM_004943583		Uncharacterized protein [Source:UniProtKB/TrEMBL;Acc:F1NE05] [ENSGALT00000032948]
A_87_P061136	1.953	2.291	2.139	LOC101751150	XR_140330	Gga.499	Uncharacterized protein [Source:UniProtKB/TrEMBL;Acc:H9L3I9] [ENSGALT00000040574]
A_87_P013326	-1.482	-1.653	-2.636	LOC101751760	CR391626	Gga.5687	kinesin family member 2C [Source:HGNC Symbol;Acc:6393] [ENSGALT00000016462]
A_87_P160658	1.222	-2.038	1.148	LOC419888	XR_140333	Gga.25811	Uncharacterized protein [Source:UniProtKB/TrEMBL;Acc:F1NXG6] [ENSGALT00000003400]
A_87_P026105	-1.553	-2.379	-2.989	LOC421123	BU461157	Gga.24470	Uncharacterized protein [Source:UniProtKB/TrEMBL;Acc:R4GK51] [ENSGALT00000044567]
A_87_P023754	-1.665	-2.843	-3.274	LOC421560	BX931884	Gga.9648	Gallus gallus finished cDNA, clone ChEST804b19. [BX931884]
A_87_P014489	2.148	2.390	1.342	LOC422872	CR389926	Gga.18198	Gallus gallus finished cDNA, clone ChEST916j2. [CR389926]
A_87_P013792	2.659	-1.087	-1.369	LOC422887	CR390999	Gga.55302	Gallus gallus finished cDNA, clone ChEST738p23. [CR390999]
A_87_P143453	3.351	-1.128	-1.328	LOC422887	CR390999	Gga.55302	Gallus gallus finished cDNA, clone ChEST738p23. [CR390999]
A_87_P317967	1.161	-2.206	1.291	LOC424523	XM_422360		Uncharacterized protein [Source:UniProtKB/TrEMBL;Acc:F1P490] [ENSGALT00000010222]
A_87_P107888	-2.070	1.083	-1.236	LOC425137	NM_001278080	Gga.38962	Gallus gallus aldo-keto reductase family 1, member B1-like (LOC425137), mRNA [NM_001278080]
A_87_P013636	1.847	1.884	2.368	LOC426456	CR391215	Gga.18678	Gallus gallus finished cDNA, clone ChEST206b13. [CR391215]
A_87_P068181	2.002	-1.257	1.788	LOC427773	XM_425344		ectonucleoside triphosphate diphosphohydrolase 2 [Source:HGNC Symbol;Acc:3364] [ENSGALT00000040222]
A_87_P023856	-1.511	-2.008	-1.410	LOC768911	BX931610	Gga.7559	Gallus gallus finished cDNA, clone ChEST400f11. [BX931610]
A_87_P055616	-1.462	-2.066	-1.262	LPAR1	NM_001115082	Gga.9756	Gallus gallus lysophosphatidic acid receptor 1 (LPAR1), mRNA [NM_001115082]
A_87_P080671	-1.073	-2.017	1.309	LRRC18	XM_004942134		leucine rich repeat containing 18 [Source:HGNC Symbol;Acc:23199] [ENSGALT00000009958]
A_87_P302368	1.581	-2.088	1.435	LRRC72	XM_418694		leucine rich repeat containing 72 [Source:HGNC Symbol;Acc:42972] [ENSGALT00000017565]
A_87_P115443	2.175	1.083	-1.048	MAPT	NM_001199193	Gga.15322	Gallus gallus microtubule-associated protein tau (MAPT), mRNA [NM_001199193]
A_87_P199993	-1.662	-1.350	-2.342	MB	NM_001167752	Gga.1960	Gallus gallus myoglobin (MB), mRNA [NM_001167752]
A_87_P011904	-1.388	-2.601	-3.484	MED22	CR523011	Gga.42364	Gallus gallus finished cDNA, clone ChEST795b24. [CR523011]
A_87_P251453	-1.549	-3.262	-5.174	MED22	CR523011	Gga.42364	Gallus gallus finished cDNA, clone ChEST795b24. [CR523011]
A_87_P312677	-1.116	-1.553	-2.039	MED22	NM_001045830	Gga.42364	Gallus gallus mediator complex subunit 22 (MED22), mRNA [NM_001045830]
A_87_P011656	-1.081	-1.328	-2.628	MEGF11	CR523376	Gga.11013	Gallus gallus finished cDNA, clone ChEST876f13. [CR523376]
A_87_P062896	-1.388	-2.819	1.052	MEOX1	NM_204765	Gga.288	Gallus gallus mesenchyme homeobox 1 (MEOX1), mRNA [NM_204765]
A_87_P013958	-1.957	-2.658	-1.244	MOGAT2	CR390740	Gga.37179	Uncharacterized protein [Source:UniProtKB/TrEMBL;Acc:F1NZ99] [ENSGALT00000022919]
A_87_P121398	-1.007	1.919	3.405	MRGPRH	CR385967	Gga.15061	Gallus gallus finished cDNA, clone ChEST239e19. [CR385967]
A_87_P024179	-1.911	-3.490	-1.400	MRRF	BX930788	Gga.7262	mitochondrial ribosome recycling factor [Source:HGNC Symbol;Acc:7234] [ENSGALT00000002038]

A_87_P008812	1.295	2.083	1.309	MST1	NM_205213	Gga.670	Gallus gallus macrophage stimulating 1 (hepatocyte growth factor-like) (MST1), mRNA [NM_205213]
A_87_P075246	-2.175	2.108	-3.131	MVD	CR338929	Gga.48999	mevalonate (diphospho) decarboxylase [Source:HGNC Symbol;Acc:7529] [ENSGALT00000011769]
A_87_P200998	-2.184	1.015	-2.615	MVD	CR338929	Gga.48999	mevalonate (diphospho) decarboxylase [Source:HGNC Symbol;Acc:7529] [ENSGALT00000011769]
A_87_P009195	-2.123	-1.025	-1.458	MYH15	NM_204766	Gga.2617	Gallus gallus myosin, heavy chain 15 (MYH15), mRNA [NM_204766]
A_87_P108978	-2.049	1.055	-1.319	MYH15	NM_204766	Gga.2617	Gallus gallus myosin, heavy chain 15 (MYH15), mRNA [NM_204766]
A_87_P053486	-1.701	-2.199	-2.175	MYL1	NM_001044632	Gga.4835	Gallus gallus myosin, light chain 1, alkali; skeletal, fast (MYL1), mRNA [NM_001044632]
A_87_P009549	-1.631	-3.131	-2.682	MYL4	J02823	Gga.21937	myosin, light chain 4, alkali; atrial, embryonic [Source:HGNC Symbol;Acc:7585] [ENSGALT00000000816]
A_87_P157248	-1.673	-3.265	-2.888	MYL4	J02823	Gga.21937	myosin, light chain 4, alkali; atrial, embryonic [Source:HGNC Symbol;Acc:7585] [ENSGALT00000000816]
A_87_P023086	-1.984	-1.967	-3.316	MYO3A	BX933331	Gga.10560	Gallus gallus finished cDNA, clone ChEST192m15. [BX933331]
A_87_P095916	-2.021	-1.245	1.014	NHEDC2	CR387753	Gga.16446	Uncharacterized protein [Source:UniProtKB/TrEMBL;Acc:E1C3V4] [ENSGALT00000020128]
A_87_P024019	-1.611	-1.801	-2.457	NMNAT3	BX931202	Gga.11021	nicotinamide nucleotide adenylyltransferase 3 [Source:HGNC Symbol;Acc:20989] [ENSGALT00000046024]
A_87_P110323	1.668	-1.335	-2.185	NMS	XM_004938552		Uncharacterized protein [Source:UniProtKB/TrEMBL;Acc:F1NR12] [ENSGALT00000041350]
A_87_P144413	1.236	-2.343	1.429	NR1I3	NM_204702	Gga.237	Gallus gallus nuclear receptor subfamily 1, group I, member 3 (NR1I3), mRNA [NM_204702]
A_87_P124353	1.566	2.115	1.470	NRAS	NM_001012549	Gga.22051	Gallus gallus neuroblastoma RAS viral (v-ras) oncogene homolog (NRAS), mRNA [NM_001012549]
A_87_P226148	1.702	-2.142	1.576	NT5C1A	BU411420	Gga.8058	5'-nucleotidase, cytosolic IA [Source:HGNC Symbol;Acc:17819] [ENSGALT00000006051]
A_87_P011916	-1.222	2.163	1.539	ORAOV1	NM_001031186	Gga.22117	Gallus gallus oral cancer overexpressed 1 (ORAOV1), mRNA [NM_001031186]
A_87_P055531	-1.175	2.049	1.592	ORAOV1	NM_001031186	Gga.22117	Gallus gallus oral cancer overexpressed 1 (ORAOV1), mRNA [NM_001031186]
A_87_P129473	-1.221	-1.384	-2.317	OSTN	NM_001098608	Gga.13448	Gallus gallus osteocrin (OSTN), mRNA [NM_001098608]
A_87_P037755	-2.508	-1.988	-3.200	OTOR	NM_204732	Gga.263	Gallus gallus otoraplin (OTOR), mRNA [NM_204732]
A_87_P034873	1.630	2.169	1.334	OVALX	NM_001276386		Gallus gallus ovalbumin-related protein X (SERPINB14C) (OVALX), mRNA [NM_001276386]
A_87_P065251	-2.251	1.036	-1.466	PADI1	AB842273		Peptidylarginine deiminase type I; Uncharacterized protein [Source:UniProtKB/TrEMBL;Acc:E1C0W6] [ENSGALT00000000655]
A_87_P097016	-1.357	-1.878	-2.084	PAPOLG	XM_004940117		poly(A) polymerase gamma [Source:HGNC Symbol;Acc:14982] [ENSGALT00000012751]
A_87_P080016	2.084	-1.480	1.246	PCDH15	DQ354419	Gga.27533	Gallus gallus protocadherin-15-CD1 isoform 1 mRNA, complete cds. [DQ354419]
A_87_P019799	-1.422	-1.684	-2.481	PCP4	NM_001278148	Gga.7397	Gallus gallus Purkinje cell protein 4 (PCP4), mRNA [NM_001278148]
A_87_P175983	2.135	2.432	1.314	PDC	XM_004943303		phosducin [Source:HGNC Symbol;Acc:8759] [ENSGALT00000008131]
A_87_P038145	2.975	-2.484	3.053	PIT54	NM_207180	Gga.1164	Gallus gallus PIT54 protein (PIT54), mRNA [NM_207180]
A_87_P055601	1.067	-2.175	1.413	PLP1	NM_205277	Gga.1803	Gallus gallus proteolipid protein 1 (PLP1), mRNA [NM_205277]
A_87_P095531	2.157	1.526	1.160	PPM1K			Uncharacterized protein [Source:UniProtKB/TrEMBL;Acc:F1P138] [ENSGALT00000018387]

A_87_P093606	-1.271	3.107	1.395	PRDM6	XM_424413		PR domain containing 6 [Source:HGNC Symbol;Acc:9350] [ENSGALT00000008588]
A_87_P170038	1.736	2.384	1.144	PRDX4	DR417601	Gga.34481	gPGC_EST07443 Embryonic gonadal PGC cDNA Library Gallus gallus cDNA 5', mRNA sequence [DR417601]
A_87_P063281	-2.190	1.477	1.514	PTPN7	XM_001232668	Gga.28324	protein tyrosine phosphatase, non-receptor type 7 [Source:HGNC Symbol;Acc:9659] [ENSGALT00000000839]
A_87_P270878	-1.233	-2.156	-1.331	RBMX	NM_001079728	Gga.21807	Gallus gallus RNA binding motif protein, X-linked (RBMX), mRNA [NM_001079728]
A_87_P116733	-1.265	-2.130	1.228	RFXDC2	BX934393	Gga.12663	regulatory factor X, 7 [Source:HGNC Symbol;Acc:25777] [ENSGALT00000001028]
A_87_P078541	-1.141	-2.115	-1.029	RGS16	NM_001277544		Gallus gallus regulator of G-protein signaling 16 (RGS16), mRNA [NM_001277544]
A_87_P104343	-1.774	-3.067	1.390	RGS20	NM_204842	Gga.352	Gallus gallus regulator of G-protein signaling 20 (RGS20), mRNA [NM_204842]
A_87_P154873	1.753	2.005	1.542	RNF152	XM_004939697		E3 ubiquitin-protein ligase RNF152 [Source:UniProtKB/Swiss-Prot;Acc:E1C2W7] [ENSGALT00000043782]
A_87_P146108	2.113	2.335	1.383	RPL6	DR416206	Gga.3486	gPGC_EST06048 Embryonic gonadal PGC cDNA Library Gallus gallus cDNA 5', mRNA sequence [DR416206]
A_87_P070641	-2.271	-1.714	-2.211	RSPO4	AB690565	Gga.55711	R-spondin 4 [Source:HGNC Symbol;Acc:16175] [ENSGALT00000039389]
A_87_P117374	1.572	-2.118	-1.266	RSRP1	BX935301	Gga.1137	Gallus gallus finished cDNA, clone ChEST959g23. [BX935301]
A_87_P009018	-1.210	-2.016	-1.424	S100A11	NM_205166	Gga.639	Gallus gallus S100 calcium binding protein A11 (S100A11), mRNA [NM_205166]
A_87_P056551	-1.212	-2.061	-1.466	S100A11	NM_205166	Gga.639	Gallus gallus S100 calcium binding protein A11 (S100A11), mRNA [NM_205166]
A_87_P064056	1.696	2.009	1.029	SCMH1	XM_001232076		sex comb on midleg homolog 1 (Drosophila) [Source:HGNC Symbol;Acc:19003] [ENSGALT00000000949]
A_87_P095371	-2.113	-1.053	-1.644	SCRG1	XM_420523		stimulator of chondrogenesis 1 [Source:HGNC Symbol;Acc:17036] [ENSGALT00000017493]
A_87_P011946	1.208	-2.317	1.767	SDC3	CR522958	Gga.20152	Gallus gallus syndecan 3 (SDC3), mRNA. [Source:RefSeq mRNA;Acc:NM_205383] [ENSGALT00000000793]
A_87_P021044	2.006	-2.262	1.801	SEMA5B	XM_422096	Gga.31259	sema domain, seven thrombospondin repeats (type 1 and type 1-like), transmembrane domain (TM) and short cytoplasmic domain, (semaphorin) 5B [Source:HGNC Symbol;Acc:10737] [ENSGALT00000019105]
A_87_P138443	2.123	1.153	-1.681	SERPINH1	NM_205291	Gga.4168	Gallus gallus serpin peptidase inhibitor, clade H (heat shock protein 47), member 1, (collagen binding protein 1) (SERPINH1), mRNA [NM_205291]
A_87_P216583	2.261	1.073	-1.461	SERPINH1	NM_205291	Gga.4168	Gallus gallus serpin peptidase inhibitor, clade H (heat shock protein 47), member 1, (collagen binding protein 1) (SERPINH1), mRNA [NM_205291]
A_87_P093966	1.149	2.018	1.181	SESN2	BU407331	Gga.55126	Uncharacterized protein [Source:UniProtKB/TrEMBL;Acc:F1NGI3] [ENSGALT00000009061]
A_87_P071421	-1.378	-2.171	-1.272	SHMT1	XM_004945202		serine hydroxymethyltransferase 1 (soluble) [Source:HGNC Symbol;Acc:10850] [ENSGALT00000008093]
A_87_P023775	1.412	2.344	1.110	SLC5A11	BX931813	Gga.50802	solute carrier family 5 (sodium/inositol cotransporter), member 11 [Source:HGNC Symbol;Acc:23091] [ENSGALT00000009548]
A_87_P021800	-1.501	-1.426	-2.403	SNCA	NM_204673	Gga.2726	Gallus gallus synuclein, alpha (non A4 component of amyloid precursor) (SNCA), mRNA [NM_204673]
A_87_P037801	1.336	2.112	1.411	SOX14	NM_204761	Gga.286	Gallus gallus SRY (sex determining region Y)-box 14 (SOX14), mRNA [NM_204761]
A_87_P086866	-2.388	1.121	-1.859	SPEG	BU236587	Gga.27820	SPEG complex locus [Source:HGNC Symbol;Acc:16901] [ENSGALT00000043042]

A_87_P031536	-1.014	-2.058	-2.041	SPTBN5	BU255435	Gga.6162	spectrin, beta, non-erythrocytic 5 [Source:HGNC Symbol;Acc:15680] [ENSGALT00000014533]
A_87_P014649	-1.207	2.061	1.198	SRSF5	NM_001031197	Gga.4502	Gallus gallus serine/arginine-rich splicing factor 5 (SRSF5), mRNA [NM_001031197]
A_87_P124158	-1.212	2.219	1.253	SRSF5	NM_001031197	Gga.4502	Gallus gallus serine/arginine-rich splicing factor 5 (SRSF5), mRNA [NM_001031197]
A_87_P079586	3.140	1.945	2.109	TACSTD2	NM_001277676		Gallus gallus tumor-associated calcium signal transducer 2 (TACSTD2), mRNA [NM_001277676]
A_87_P098601	-1.778	-1.869	-2.999	TCP10	XM_419608		Uncharacterized protein [Source:UniProtKB/TrEMBL;Acc:F1NVN7] [ENSGALT00000018514]
A_87_P098116	-2.103	1.058	-1.382	TCTE1	XM_004935389		t-complex-associated-testis-expressed 1 [Source:HGNC Symbol;Acc:11693] [ENSGALT00000016530]
A_87_P003295	1.170	1.399	2.496	TIMM50	XM_001233750		translocase of inner mitochondrial membrane 50 homolog (S. cerevisiae) [Source:HGNC Symbol;Acc:23656] [ENSGALT00000044658]
A_87_P132678	1.142	3.762	1.666	TLR1LA	NM_001007488	Gga.22623	Gallus gallus toll-like receptor 1-like A (TLR1LA), mRNA [NM_001007488]
A_87_P129588	1.537	2.529	1.393	TLR4	NM_001030693	Gga.32116	Gallus gallus toll-like receptor 4 (TLR4), mRNA [NM_001030693]
A_87_P170453	-2.036	-1.313	-1.516	TMEM194A	XM_001232566		transmembrane protein 194A [Source:HGNC Symbol;Acc:29001] [ENSGALT00000000664]
A_87_P064916	1.283	-2.107	1.584	TRIM29	XM_417892		tripartite motif containing 29 [Source:HGNC Symbol;Acc:17274] [ENSGALT00000010874]
A_87_P024599	-1.442	4.836	-1.586	TRIM65	BX929756	Gga.10224	Gallus gallus finished cDNA, clone ChEST791p15. [BX929756]
A_87_P035266	-1.141	1.465	-2.063	TRPC3	AY130981	Gga.106	Gallus gallus transient receptor potential protein mRNA, partial cds. [AY130981]
A_87_P265968	-2.034	1.205	-1.690	TSTA3	NM_001277448	Gga.48657	Gallus gallus tissue specific transplantation antigen P35B (TSTA3), mRNA [NM_001277448]
A_87_P071201	-1.217	-1.592	-2.204	UNCX	XM_414766		UNC homeobox [Source:HGNC Symbol;Acc:33194] [ENSGALT00000041002]
A_87_P035138	-1.122	-2.160	-1.049	UTS2	NM_206990	Gga.14388	Gallus gallus urotensin 2 (UTS2), mRNA [NM_206990]
A_87_P067151	-2.158	1.034	-1.535	UTS2R	XM_004946093		urotensin 2 receptor [Source:HGNC Symbol;Acc:4468] [ENSGALT00000002482]
A_87_P036253	2.844	-1.179	1.562	XPO1	AJ720676	Gga.24191	Gallus gallus mRNA for hypothetical protein, clone 23d16. [AJ720676]
A_87_P065011	1.341	2.053	1.150	ZBTB16	CR523202	Gga.17584	zinc finger and BTB domain containing 16 [Source:HGNC Symbol;Acc:12930] [ENSGALT00000011346]

[#] For comparison between the CTL and heat-stressed groups, gene expressions with more than two-fold changes (up or down) were considered as significantly different ($P < 0.05$).

Supplementary Table S2. The differentially expressed proteins in SYFs of layer-type L2 strain Taiwan country chickens after acute heat stress.

Spot No.	Average ratio[a] R0/C	R2/C	R6/C	Protein identity	GenBank accession	Gene symbol	MALDI-MS PMF[b]	Theoretical Mr/pI	Score (TOF/TOF-TOF)	MALDI-MS/MS LIFT	Peptide
52	-1.43	1.70	1.37	heat shock protein 108	gi|211943	HSP108	34/185 (46)	91726/4.68	131/397		
95	1.13	1.62	1.84	vitellogenin I	gi|1871444	VTG1	44/219 (27)	212608/9.9	76/57	1 (57)	R.YLLDLLPAAASHR.S
129	-1.14	1.58	1.35	Chain A, Crystal Structure Of Aluminum-Bound Ovotransferrin At 2.15 Angstrom Resolution	gi|83754919		29/134 (57)	77518/6.8	185/96	2 (67, 54)	R.SAGWNIPIGTLIHR.G
162	1.25	1.62	1.53	vitellogenin I	gi|1871444	VTG1	45/205 (28)	212608/9.9	105/124	2 (89, 61)	R.YLLDLLPAAASHR.S
182	-1.40	2.30	1.49	78 kDa glucose-regulated protein precursor	gi|45382769	GRP78	36/206 (53)	72088/4.98	196/450	4 (87, 151, 154, 145)	R.ITPSYVAFTPEGER.L
184	-1.29	2.13	1.51	78 kDa glucose-regulated protein precursor	gi|45382769	GRP78	8/11 (47)	72088/4.98	174/367	4 (67, 78, 130, 182)	R.VEIIANDQGNR.I
192	-1.22	1.58	1.06	lamin-A	gi|45384214	LOC396224	26/152 (41)	73348/6.52	136/NS	NS	R.AVPATEFVTTAVLPEER.T
194	-1.21	1.55	1.42	heat shock cognate 70	gi|2996407	hsc70	24/195 (47)	71868/5.42	79/54	1 (54)	R.FEELNADLFR.G
199	-1.13	1.51	1.23	serum albumin precursor	gi|45383974	ALB	24/157 (65)	71868/5.42	65/NS	NS	K.NQGSWGTGGNIR.T
200	-1.08	2.06	1.55	heat shock cognate 70	gi|2996407	hsc70	24/163 (45)	71077/5.35	95/160	2 (26, 161)	R.TTPSYVAFTDTER.L
206	1.16	1.86	1.38	heat shock protein 70	gi|421931777	HSP70	23/177 (40)	70098/5.55	68/60	2 (57, 30)	R.FEELNADLFR.G
209	1.32	1.68	1.30	Chain N, Avian Mitochondrial Respiratory Complex Ii At 1.8 Angstrom Resolution	gi|110590578		26/224 (58)	69098/6.2	111/173	3 (59, 87, 85)	R.TGHSLLHTLYGR.S
213	-1.15	2.05	1.47	heat shock cognate 70	gi|2996407	hsc70	30/163 (50)	71011/5.35	129/215	3 (43, 61, 167)	R.TTPSYVAFTDTER.L
216	-1.03	1.51	1.39	heat shock protein 75 kDa, mitochondrial	gi|57525126	TRAP1	31/200 (44)	80082/6.7	102/103	3 (51, 38, 73)	R.FIAQAYDKPR.Y
220	1.25	1.87	1.41	heat shock cognate 70	gi|2996407	hsc70	16/173 (36)	71011/5.35	75/63	1 (63)	R.FEELNADLFR.G
221	1.08	2.24	1.57	heat shock protein 70	gi|421931777	HSP70	37/208 (59)	70098/5.5	140/172	3 (80, 69, 86)	R.FEELNADLFR.G
235	1.06	2.27	1.52	heat shock protein 70	gi|421931777	HSP70	21/73 (34)	70098/5.5	165/139	3 (47, 29, 116)	R.ARFEELNADLFR.G
239	-1.15	1.88	1.50	heat shock protein 70	gi|421931777	HSP70	23/185 (35)	70098/5.5	93/79	1 (79)	K.GQIQEIVLVGGSTR.I
248	-1.06	1.91	1.48	stress-70 protein, mitochondrial precursor		HSPA9	28/174 (48)	73432/6.05	114/134	3 (63, 55, 76)	K.VQQTVQDLFGR.A
253	-1.13	2.19	1.64	stress-70 protein, mitochondrial precursor	gi|57524986	HSPA9	25/92 (47)	73432/6	154/115	3 (5, 79, 67)	R.TTPSVVAFTADGER.L
282	-1.08	1.79	1.66	EH domain-containing protein 3	gi|71895641	EHD3	33/167 (63)	60914/6.2	152/128	2 (69, 89)	K.LNAFGNAFLNR.F
289	-1.04	1.74	1.36	vitellogenin	gi|50582493	VTG	33/105 (32)	163656/10.1	120/NS	NS	R.VDGPILIFFADYR.I
293	-1.10	1.94	1.38	T-complex protein 1 subunit epsilon	gi|60302774	CCT5	30/199 (64)	60190/5.4	102/117	3 (65, 60, 52)	R.SLHDALCVIR.N

300	1.66	2.14	1.53	60 kDa heat shock protein, mitochondrial precursor	gi|61098372	HSPD1	25/201 (43)	61105/5.6	92/320	3 (99, 143, 139)	R.AAVEEGIVPGGGCALLR.C
302	-1.12	1.50	1.40	ankyrin 3, node of Ranvier (ankyrin G)	gi|459642348	ANK3	44/111 (14)	477667/6	74/NS	NS	R.LYGPTNFSPIINHVAR.F
303	-1.02	1.54	1.34	PREDICTED: cytoskeleton-associated protein 4, partial	gi|513159594	CKAP4	9/28 (21)	54564/4.8	77/125	2 (28, 98)	K.FNSVEGSVDELR.S
312	1.33	2.45	1.71	60 kDa heat shock protein, mitochondrial precursor	gi|61098372	HSPD1	31/187 (55)	61105/5.6	136/396	3 (121, 135, 203)	R.AAVEEGIVPGGGCALLR.C
319	-1.18	2.57	1.77	60 kDa heat shock protein, mitochondrial precursor	gi|61098372	HSPD1	35/194 (61)	61105/5.6	183/431	3 (132, 159, 202)	R.AAVEEGIVPGGGCALLR.C
330	-1.17	1.53	1.36	apolipoprotein B	gi|102221132	APOB	81/222 (22)	524519/8.99	83/204	3 (72, 61, 126)	R.IPHTVTAPTFGK.L
333	-1.26	1.55	1.45	apolipoprotein B	gi|102221132	APOB	61/144 (16)	524519/8.99	79/64	2 (62, 25)	R.IPHTVTAPTFGK.L
341	-1.18	1.70	1.43	T-complex protein 1 subunit eta	gi|71895883	CCT7	28/210 (58)	60881/5.9	92/148	2 (79, 70)	K.LPIGDVATQYFADR.D
353	1.1	1.9	1.35	T-complex protein 1 subunit theta	gi|52138673	CCT8	33/156 (51)	60017/5.24	167/109	2 (100, 41)	K.HYSGLEEAVYR.N
362	-1.13	1.82	1.36	T-complex protein 1 subunit alpha	gi|57530301	TCP1	35/101 (63)	61056/5.6	136/150	3 (43, 38, 119)	K.FATEAAITILR.I
372	1.00	1.81	1.54	T-complex protein 1 subunit beta	gi|60302718	CCT2	37/169 (65)	57737/5.9	180/76	2 (43, 61)	K.IHPQTIIAGWR.A
382	-1.32	2.07	1.69	protein disulfide-isomerase A3 precursor	gi|45383890	PDIA3	30/145 (51)	56546/5.68	195/114	3 (21, 34, 112)	K.FVMQEEFSR.D
398	-1.26	2.39	1.83	protein disulfide-isomerase A3 precursor	gi|45383890	PDIA3	30/170 (55)	56546/5.68	196/138	3 (56, 29, 101)	K.FVMQEEFSR.D
402	1.73	2.08	1.58	Chain E, Crystal Structure Of Native Chicken Fibrinogen With Two Different Bound Ligands	gi|21730887		30/189 (67)	53400/7.7	123/NS	NS	K.EDGGGWWYNR.C
406	1.46	2.02	1.45	PREDICTED: A-kinase anchor protein 9 isoform X22	gi|513193274	PCNT	48/153 (15)	481664/5	86/NS	NS	R.ELGLQEDNPGVYNGR.W
409	1.48	1.64	1.34	fibrinogen beta chain, partial	gi|211780		24/181 (60)	53272/7.7	132/152	3 (45, 90, 74)	K.EDGGGWWYNR.C
411	1.02	1.89	1.51	PREDICTED: aldehyde dehydrogenase, mitochondrial isoform X3	gi|513210248	ALDH2	27/202 (56)	60459/8.5	134/130	2 (79, 79)	K.TIPLDGDFFCYTR.H
412	1.25	2.04	1.60	fibrinogen beta chain, partial	gi|211780		27/193 (66)	53272/7.69	106/83	2 (41, 69)	K.EDGGGWWYNR.C
415	1.07	2.17	1.63	tubulin alpha	gi|223280		17/165 (50)	46259/4.86	133/273	3 (126, 35, 171)	R.AVFVDLEPTVIDEVR.T
418	1.41	1.66	1.48	recombination activating protein 1	gi|4973167	RAG-1	21/162 (22)	112660/9.6	60/NS	NS	K.EDGGGWWYNR.C
426	1.08	1.86	1.55	RecName: Full=Tubulin alpha-1 chain	gi|135393	TUBA1C	19/142 (51)	46259/4.86	156/126	2 (126, 19)	R.AVFVDLEPTVIDEVR.T
427	-1.05	2.16	1.63	RecName: Full=Tubulin alpha-1 chain	gi|135393	TUBA1C	17/158 (47)	46259/4.86	132/223	3 (107,129,44)	R.LIGQIVSSITASLR.F
430	1.31	1.87	1.66	PREDICTED: cytoskeleton-associated protein 4, partial	gi|513159594	CKAP4	26/175 (56)	54564/4.8	101/173	3 (80, 55, 102)	K.FNSVEGSVDELR.S

											R.SYELAGGSLGGLLEALDSMGLR.G
433	-1.3	1.82	1.81	tubulin beta-3 chain	gi|153792017	TUBB4B	17/87 (30)	50285/4.6	90/NS	NS	+ Oxidation (M)
434	-1.25	1.82	1.54	keratin, type II cytoskeletal cochleal	gi|45384378	KRT7	36/135 (57)	53770/5.86	215/131	3 (12, 102, 58)	R.ISLGGSSSYSVR.S
436	-1.14	1.72	1.63	PREDICTED: keratin, type II cytoskeletal 8	gi|513233477	KRT8	26/128 (63)	40680/4.8	138/133	3 (64, 61, 73)	R.GLFEGYVGTLR.R
439	-1.10	1.71	1.49	keratin, type II cytoskeletal cochleal	gi|45384378	KRT7	31/144 (55)	53770/5.9	153/67	2 (67, 4)	K.VDALTDEINFLR.S
440	-1.03	1.69	1.35	selenium-binding protein 1	gi|480540318	SELENBP1	19/198 (48)	53098/6.2	84/89	1 (66)	K.LVGQVFLGGSIVR.G
445	-1.02	2.04	1.42	succinyl-CoA:3-ketoacid-coenzyme A transferase 1, mitochondrial	gi|60592998	OXCT1	12/84 (36)	56549/8.9	44/30	1 (30)	R.MVSSYVGENAEFER.Q
446	1.08	1.80	1.43	ATP synthase subunit alpha, mitochondrial isoform	gi|363745506	ATP5A1W	18/183 (41)	60162/9.5	76/90	2 (19, 90)	K.GIRPAINVGLSVSR.V
449	-1.15	2.02	1.75	PREDICTED: aldehyde dehydrogenase, mitochondrial isoform X3	gi|513210248	ALDH2	23/ 174 (49)	60459/8.5	122/134	2 (71, 91)	K.TIPLDGDFFCYTR.H
452	-1.34	1.87	1.36	vimentin	gi|114326309	VIM	41/156 (62)	53167/4.94	166/119	3 (31, 84, 59)	R.YSLGSALRPSSAR.Y
459	-1.22	1.91	1.42	desmin	gi|2959450	DES	29/163 (59)	51687/5.2	173/187	3 (62, 73, 114)	R.TSAVPTLSTFR.T
460	-1.02	1.81	1.66	PREDICTED: keratin, type II cytoskeletal 8	gi|513233477	KRT8	22/150 (55)	40680/4.8	55/183	4 (75, 46, 82, 77)	R.GLFEGYVGTLR.R
463	-1.02	1.93	1.69	PREDICTED: aldehyde dehydrogenase, mitochondrial isoform X3	gi|513210248	ALDH2	26/140 (49)	60459/8.5	92/158	3 (19, 97, 89)	K.DIDKANYVSQALR.A
464	1.04	1.77	1.49	PREDICTED: ATP synthase subunit alpha, mitochondrial isoform X2	gi|363745506	ATP5A1W	25/155 (45)	60162/9.5	123/139	3 (3, 109, 61)	K.GIRPAINVGLSVSR.V
470	1.18	1.87	1.24	PREDICTED: ATP synthase subunit alpha, mitochondrial isoform X2	gi|363745506	ATP5A1W	22/174 (37)	60263/9.73	97/117	2 (89, 60)	R.EAYPGDVFYLHSR.L
471	-1.46	1.61	1.34	desmin	gi|2959450	DES	25/158 (54)	51689/5.2	182/252	4 (64, 84, 66, 129)	R.TSAVPTLSTFR.T
472	-1.24	1.38	1.56	hypothetical protein RCJMB04_32b9	gi|53136648		16/161 (39)	49428/5.35	86/90	2 (49, 66)	R.DFLLKPELLR.A
486	-1.05	2.00	1.48	tubulin alpha	gi|223280		16/118 (49)	46259/4.9	93/110	3 (59, 84, 21)	K.HQSLGGQYGVR.G
487	-1.22	2.40	1.80	phosphatidylinositol-4,5-bisphosphate 3-kinase catalytic subunit gamma isoform	gi|313747561	PIK3CG	21/109 (29)	129184/6.8	66/NS	NS	R.QSMEEVDLKLR.G + Oxidation (M)
496	-1.05	1.85	1.52	tubulin beta-2 chain	gi|52138699	TUBB2B	33/187 (76)	50377/4.64	204/251	4 (65, 39, 38, 196)	R.ISEQFTAMFR.R
500	-1.19	1.97	1.54	tubulin beta-7 chain	gi|45384338	TUBB2A	34/197 (81)	50095/4.64	218/260	3 (64, 34, 58, 196)	R.ISEQFTAMFR.R
503	-1.00	1.81	1.70	PREDICTED: septin-11 isoform X2	gi|513183226	SEPT11	14/130 (37)	51036/6.4	84/94	3 (45, 27, 79)	R.SYELQESNVR.L
517	-1.26	2.13	1.71	serum albumin precursor	gi|45383974	ALB	32/175 (54)	71868/5.42	96/86	2 (72, 40)	R.VSFLGHFIYSVAR.R

521	-1.23	1.72	1.31	ATP synthase subunit beta, mitochondrial precursor	gi|82082513	ATP5B	35/186 (74)	56650/5.5	214/298	4 (117, 72, 73, 133)	K.AHGGYSVFAGVGER.T
522	-1.32	2.22	1.77	ATP synthase subunit beta, mitochondrial precursor	gi|82082513	ATP5B	37/174 (76)	56650/5.5	276/255	4 (97, 78, 51, 121)	K.AHGGYSVFAGVGER.T
553	-1.17	1.61	1.29	vitellogenin	gi|50582493	VTG	45/196 (35)	163656/10.1	120/33	1 (33)	R.FLEVVQLCR.I
554	-1.30	2.08	1.37	heat shock protein 47	gi|63511	SERPINH1	30/185 (55)	45711/9.23	118/66	1 (66)	R.LYGPASINFADDFVK.N
557	-1.24	2.13	1.46	heat shock protein 47	gi|63511	SERPINH1	34/203 (65)	45711/9.23	124/71	1 (71)	R.LYGPASINFADDFVK.N
566	-1.26	2.12	1.46	heat shock protein 47	gi|63511	SERPINH1	24/115 (49)	45711/9.23	102/85	2 (8, 85)	R.SYTVGVPMMHR.T + 2 Oxidation (M)
568	-1.08	1.55	1.39	alpha-enolase	gi|46048768	ENO1	20/187 (50)	47617/6.16	99/216	3 (73, 62, 144)	K.VVIGMDVAASEFYR.D
572	1.18	1.86	1.38	heat shock protein 47	gi|63511	Hsp47	29/169 (61)	45711/9.23	95/71	2 (17, 71)	R.SYTVGVPMMHR.T + Oxidation (M)
589	-1.06	1.81	1.52	PREDICTED: endoplasmic reticulum resident protein 44 isoform X4	gi|50736203	ERP44	15/150 (43)	47318/5.2	115/174	3 (24, 86, 117)	K.FRHPLLHIQK.T
590	1.02	1.83	1.52	thioredoxin domain-containing protein 5 precursor	gi|57530789	TXNDC5	19/177 (43)	47249/5.5	103/225	3 (80, 82, 116)	R.DLESLHSFVLR.Q
596	1.12	1.64	1.42	vimentin	gi|114326309	VIM	34/134 (60)	53167/4.94	122/46	1 (46)	R.INMPIPTFASLNLR.E + Oxidation (M)
599	-1.18	1.83	1.47	vimentin	gi|114326309	VIM	31/153 (54)	53167/4.94	111/30	1 (30)	R.INMPIPTFASLNLR.E + Oxidation (M)
602	-1.25	1.96	1.44	PREDICTED: probable cation-transporting ATPase 13A3 isoform X4	gi|513199476	ATP13A3	25/167 (24)	146045/6.2	63/109	1 (109)	R.LGPHSSAASVYAGAGGSGSR.I
604	-1.17	1.59	1.40	Chain N, Chicken Cytochrome Bc1 Complex Inhibited By An Iodinated Analogue Of The Polyketide Crocacin-d	gi|196049785		24/181 (59)	49981/5.9	112/330	3 (81, 168, 142)	R.ADLASYIDTHFK.A
620	-1.13	2.12	1.82	vimentin	gi|114326309	VIM	32/178 (59)	53167/4.94	116/131	4 (54, 17, 80, 57)	K.MALDIEIATYR.K
627	1.07	1.68	1.58	vitellogenin I	gi|1871444	VTG1	39/178 (27)	212608/9.9	61/194	3 (65, 74, 118)	K.MFGQELLFGR.L
646	1.03	1.95	1.88	keratin, type I cytoskeletal 19	gi|485049501	KRT19	33/176 (69)	46067/4.8	196/236	4 (51, 52, 74, 142)	K.TKFETEQALR.M
657	-1.13	1.47	1.51	vitellogenin	gi|212881	VTG	45/199 (31)	206862/9.9	77/126	3 (21, 46, 59)	K.MFGQELLFGR.L + Oxidation (M)
661	1.17	1.57	1.43	vitellogenin I	gi|1871444	VTG1	34/174 (21)	212608/9.9	97/90	2 (73, 40)	K.VPGVTLYYQGLR.V
663	-1.14	2.02	2.29	vimentin	gi|114326309	VIM	32/176 (54)	53167/4.9	127/154	4 (65, 52, 72, 54)	R.LGDLYEEEMR.D
665	-1.35	2.04	1.78	alpha-actin	gi|211205	ASMA	24/164 (64)	42367/5.12	102/212	3 (58, 76, 137)	R.AVFPSIVGRPR.H
671	-1.35	2.03	1.75	beta-actin	gi|211237		23/172 (72)	42052/5.18	114/NS	NS	R.AVFPSIVGRPR.H

677	1.06	1.71	1.25	vitellogenin	gi|212881	VTG	39/157 (25)	206862/9.9	92/44	2 (44, 20)	K.VAGNVQAQITPSPR.S
684	1.56	1.88	1.71	vitellogenin I	gi|1871444	VTG1	40/179 (28)	212608/9.9	70/74	2 (74, 17)	K.VPGVTLYYQGLR.V
710	-1.09	1.69	1.47	vitellogenin	gi|212881	VTG	37/162 (22)	365275/4.9	63/37	2 (37, 9)	K.VAGNVQAQITPSPR.S
742	-1.07	1.61	1.34	NADH dehydrogenase [ubiquinone] 1 alpha subcomplex subunit 10, mitochondrial	gi|71895153	NDUFA10	13/156 (44)	41633/6.2	74/78	1 (78)	K.YFPEADIHYQDR.I
756	1.02	1.66	1.37	PREDICTED: macrophage-capping protein-like isoform X6	gi|363747241	CAPG	12/69 (50)	38833/5.7	84/248	1 (17)	K.SGSLFGPAPTHPGLHIWR.V
801	-1.31	1.80	1.26	PREDICTED: heterogeneous nuclear ribonucleoprotein A3-like isoform X8	gi|513193994	LOC100859627	21/118 (46)	40774/9.7	117/116	2 (52, 91)	K.IFVGGIKEDTEEYNLR.E
818	-1.23	1.68	1.32	PREDICTED: heterogeneous nuclear ribonucleoprotein A3-like isoform X8	gi|513193994	LOC100859627	19/132 (41)	40774/9.7	116/139	2 (58, 110)	K.IFVGGIKEDTEEYNLR.E
871	1.14	1.51	1.21	60S acidic ribosomal protein P0	gi|45384494	RPLP0	12/139 (56)	34435/5.64	114/205	3 (78, 94, 94)	K.IIQLLDDYPK.C
884	-1.02	2.17	1.87	glyceraldehyde-3-phosphate dehydrogenase	gi|46048961	GAPDH	19/201 (65)	35909/9.4	128/262	3 (122, 25, 115)	R.VPTPNVSVVDLTCR.L
904	-1.17	1.73	1.75	Chain L, Remodeling Of Actin Filaments By Adf Cofilin Protein	gi|364505923		22/ 196 (63)	41921/5.2	96/166	2 (60, 134)	K.IWHHTFYNELR.V
907	1.18	1.89	1.70	annexin A2	gi|45382533	ANXA2	19/116 (63)	38901/7.6	144/27	2 (28, 25)	K.GVDEVTIINILTNR.S
927	1.09	1.63	1.57	annexin A2	gi|45382533	ANXA2	20/116 (64)	38901/7.6	175/300	4 (102, 83, 107, 98)	K.GVDEVTIINILTNR.S
935	-1.05	1.63	1.24	annexin A2	gi|45382533	ANXA2	16/111 (52)	38901/7.6	90/78	1 (78)	K.GVDEVTIINILTNR.S
936	-1.16	1.75	1.41	guanine nucleotide-binding protein G(I)/G(S)/G(T) subunit beta-1	gi|392513682	GNB1	20/190 (67)	38121/5.6	97/294	4 (74, 36, 112, 159)	K.IYAMHWGTDSR.L
939	-1.57	1.21	1.04	heterogeneous nuclear ribonucleoproteins A2/B1	gi|71896753	HNRNPA2B1	16/145 (48)	37019/9.1	109/184	2 (105, 103)	R.GGGGNFGPGPGSNFR.G
941	1.01	1.63	1.44	capping protein alpha 2 isoform	gi|211423	CapZ	12/199 (67)	32939/5.47	103/287	4 (86, 149, 55, 82)	R.LLLNNDNLLR.E
981	1.04	1.60	1.46	PREDICTED: coiled-coil domain-containing protein 141 isoform X3	gi|513193898	CCDC141	43/181 (28)	191003/5.3	85/NS	NS	R.IMEGPAFNYLDAPAVR.V + Oxidation (M)
984	-1.23	1.52	1.34	guanine nucleotide-binding protein subunit beta-2-like 1	gi|52138659	GNB2L1	12/178 (66)	35511/8.88	105/96	2 (77, 44)	R.DETNYGIPQR.A
1003	-1.19	2.09	1.81	annexin A5	gi|71895873	ANXA5	24/121 (61)	36290/5.49	158/188	3 (54, 82, 113)	K.ITGETSGHFQR.L
1011	1.03	1.51	1.59	PREDICTED: LOW QUALITY PROTEIN: annexin A4-like	gi|513220828	LOC772393	19/68 (58)	35829/5.0	117/291	2 (48, 71)	K.GVSSFNPEQEAQALR.K

1024	1.32	1.61	1.37	PREDICTED: actin, cytoplasmic 2-like isoform X1	gi|363740848	LOC776816	20/178 (62)	42108/5.2	78/207	2 (100, 138)	K.QEYDESGPSIVHR.K
1040	1.32	1.54	1.46	electron-transfer-flavoprotein, alpha polypeptide	gi|71895681	ETFA	13/151 (48)	36999/9.66	103/168	2 (77, 120)	K.VLVAQHDVYK.G
1060	1.03	1.35	1.75	Mx protein	gi|146744156	Mx	12/130 (50)	80335/7.1	58/NS	NS	R.SALGHTIEDLKEALR.T
1061	1.88	1.55	1.17	PREDICTED: nebulin-related-anchoring protein isoform X2	gi|513191976	NRAP	39/151 (25)	199352/9.7	65/NS	NS	R.GLCAIAQAESLR.Y
1066	1.04	2.06	2.05	vitellogenin III, partial	gi|555477	vtgIII	11/88 (34)	30643/9.44	70/28	1 (26)	R.NEQPTGYQLVLYTDR.Q
1070	-1.11	2.06	1.70	voltage-dependent anion-selective channel protein 2	gi|46048903	VDAC2	8/79 (44)	30293/9.2	77/264	1 (126)	K.VNNSSLVGVGYTQTLRPGVK.L
1072	1.13	2.15	1.72	voltage-dependent anion-selective channel protein 2	gi|46048903	VDAC2	13/189 (58)	30293/9.2	107/333	3 (91, 173, 126)	K.WAEYGLTFTEK.W
1077	-1.15	1.22	1.53	PREDICTED: transformation/transcription domain-associated protein	gi|513207800	TRRAP	50/144 (16)	440291/9.3	58/14	1 (14)	R.EVVTQKNLSGLVPIR.D
1085	1.14	1.82	1.80	actin, cytoplasmic type 5	gi|56119084	ACTG1	14/128 (40)	42151/5.2	70/199	2 (96, 135)	K.QEYDESGPSIVHR.K
1086	1.04	1.90	1.40	vitellogenin III, partial	gi|555477	vtgIII	9/57 (25)	30643/9.44	78/70	1 (70)	R.NEQPTGYQLVLYTDR.Q
1087	1.14	1.68	1.18	endoplasmic reticulum resident protein 29 precursor	gi|444741647	ERP29	11/157 (51)	28288/8.6	73/105	1 (105)	K.ILEQGEEFAANEVVR.I
1118	1.03	1.58	1.25	proteasome subunit alpha type-5	gi|71897167	PSMA5	14/127 (53)	26601/4.6	86/115	2 (99, 47)	R.AIGSASEGAQSSLQEVYHK.S
1134	1.01	1.70	1.47	PREDICTED: peroxiredoxin-4 isoformX2	gi|118084001	PRDX4	17/194 (72)	29843/6.3	123/193	3 (45, 82, 127)	R.QITMNDLPVGR.S
1144	-1.03	1.58	1.37	triosephosphate isomerase	gi|45382061	TPI1	18/132 (84)	26832/6.87	169/273	1 (132)	K.LSADTEVVCGAPSIYLDFAR.Q
1179	1.74	1.89	-1.02	heat shock protein 25	gi|50838655	Hsp25	NS	19086/6.4	NS/139	2 (85, 81)	R.MHLAPFASSSLATR.L
1191	-1.01	1.75	1.44	PREDICTED: thioredoxin-dependent peroxide reductase, mitochondrial isoform X7	gi|513192164	PRDX3	10/135 (58)	27595/9.9	72/191	2 (94, 130)	K.HLSINDLPVGR.S
1205	1.07	1.53	1.18	peroxiredoxin-1	gi|429836849	PRDX1	NS	22529/9.1	NS/148	3 (79, 71, 63)	R.LVQAFQFTDK.H
1209	-1.04	1.96	1.60	peroxiredoxin-1	gi|429836849	PRDX1	8/106 (53)	22529/9.1	79/180	3 (69, 64, 111)	R.LVQAFQFTDK.H
1210	1.06	2.08	1.69	peroxiredoxin-1	gi|429836849	PRDX1	NS	440291/9.3	NS/204	3 (60, 82, 120)	R.LVQAFQFTDK.H
1243	-1.32	1.65	1.35	superoxide dismutase [Mn], mitochondrial precursor	gi|45383702	SOD2	NS	25159	NS/119	1 (119)	K.HHATYVNNLNVTEEK.Y
1255	1.69	1.63	1.38	transrationally controlled tumor protein	gi|517091		11/52 (50)	19689/4.76	73/90	1 (90)	R.DCISQDEMFSDIYK.I + Oxidation (M)
1380	-1.10	1.62	1.34	PREDICTED: adenomatous polyposis coli protein isoform X1	gi|513231333	CTC-554D6.1	44/143 (18)	314856/7.7	62/247	3 (69, 68, 166)	R.IIPGFMCQGGDFTR.H

1381	-1.47	-1.65	-1.68	PREDICTED: adenomatous polyposis coli protein isoform X5	gi|513231336	CTC-554D6.1	36/124 (16)	313895/7.9	82/82	1 (82)	K.APVEQEVAIHR.I
1430	-1.07	1.63	1.45	vitellogenin	gi|212881	VTG	39/149 (24)	206862/9.9	91/NS	NS	R.VRHPATNTVVEDHVK.G
1431	-1.32	1.77	1.56	glyceraldehyde-3-phosphate dehydrogenase	gi|46048961	GAPDH	19/140 (53)	35909/9.4	97/268	3 (126, 67, 129)	R.VPTPNVSVVDLTCR.L
1432	1.33	1.92	1.49	guanine nucleotide-binding protein subunit beta-2-like 1	gi|52138659	GNB2L1	18/164 (79)	35511/8.88	157/150	2 (68, 108)	R.DETNYGIPQR.A
1434	1.04	2.15	1.56	tubulin alpha	gi|223280		16/143 (49)	46259/4.86	106/199	3 (94, 30, 133)	R.AVFVDLEPTVIDEVR.T
1435	-1.09	2.23	1.56	PREDICTED: cytoskeleton-associated protein 4, partial	gi|513159594	CKAP4	28/133 (56)	54564/4.8	148/81	2 (42, 71)	K.FNSVEGSVDELR.S
1436	-1.05	1.65	1.50	protein disulfide-isomerase precursor	gi|312283582	P4HB	19/140 (56)	57772/4.56	114/216	4 (109, 19, 94, 75)	R.AANFEQALAAHR.H
1438	-1.02	1.99	1.83	kelch-like protein 7	gi|61098073	KLHL7	17/136 (31)	67293/6.19	58/NS	NS	K.SYELPDGQVITIGNER.F
1439	1.05	1.53	1.45	SPRY domain-containing protein 7	gi|57529266	SPRYD7	13/148 (47)	22120/6.34	77/NS	NS	K.AVGISNFNHEQIER.I
1440	1.03	1.51	1.31	Chain A, Crystal Structure Of Aluminum-Bound Ovotransferrin At 2.15 Angstrom Resolution	gi|83754919		29/141 (52)	77518/6.8	153/165	3 (56,29, 134)	R.SAGWNIPIGTLIHR.G

[a] Average ratio of differential expression ($P < 0.05$) between heat-stressed TCCs with 0 hours recovery (R0)/2-hour recovery (R2)/6 hour (R6) and non–heat-stressed chickens (C) analyzed from triplicate gels. The ratio over 1.3-fold means significant difference. -, represents downregulation.

[b] The column refers to the results of the MALDI-MS PMF analysis, i.e., the number of assigned peptides and percent sequence coverage.

Abbreviations: LIFT, laser-induced forward transfer; MALDI-MS, matrix-assisted laser desorption/ionization mass spectrometer; TOF, time of flight; NS, no significant match; PMF, peptide mass fingerprinting
